# Kinetic Monte Carlo–Ising Machine Optimization for Atomistic Inverse Design of Solid Electrolytes

**DOI:** 10.1021/acs.jpclett.6c02157

**Published:** 2026-09-03

**Authors:** Ai Koizumi, Tomofumi Tada, Ryo Tamura

**Affiliations:** † Center for Basic Research on Materials, National Institute for Materials Science, 1-1 Namiki, Tsukuba, Ibaraki 305-0044, Japan; ‡ Center for Computational Sciences, 13121 University of Tsukuba, 1-1-1 Tennodai, Tsukuba, Ibaraki 305-8577, Japan; § 12923 Kyushu University Platform of Inter/Transdisciplinary Energy Research, Kyushu University, 744 Motooka, Nishi-ku, Fukuoka 819-0395, Japan; ∥ Graduate School of Frontier Sciences, The University of Tokyo, 5-1-5 Kashiwa-no-ha, Kashiwa, Chiba 277-8561, Japan

## Abstract

Maximizing ionic conductivity remains a fundamental challenge in the atomistic design of solid electrolytes. To this end, we present a framework that combines kinetic Monte Carlo (KMC) and factorization machine with quadratic-optimization annealing (FMQA), an Ising-machine-based black-box optimization algorithm for large-scale combinatorial problems. KMC evaluates the ionic conductivity for a given dopant configuration, whereas FMQA iteratively learns a surrogate model from a small configuration-conductivity data set and proposes configurations expected to maximize conductivity. To address the severe combinatorial explosion in large KMC simulation cells, we partition the configuration space for parallel optimization. As a proof of concept, we apply this KMC–FMQA framework to bulk 8 mol % yttria-stabilized zirconia, identifying a dopant configuration with an order-of-magnitude higher conductivity than that of random configurations. Combined with experiments, this framework will enable the determination of microscopic structures from measured conductivity, providing insight into the underlying transport mechanisms.

## Graphical abstract

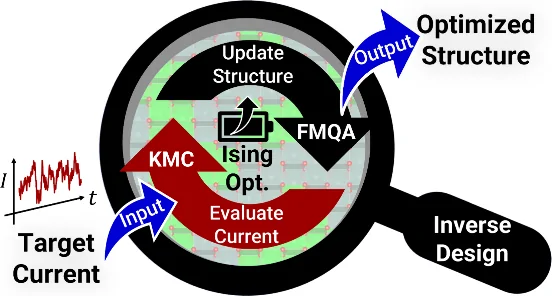

Solid oxide fuel cells (SOFCs)^1–6^ are next-generation energy conversion devices, and ionic conductivity of solid electrolytes, which are a key component of SOFCs, has been extensively investigated to improve their performance. In the design of solid electrolytes, the microscopic structure plays a crucial role in determining ionic conductivity, especially when SOFC devices are miniaturized. For example, yttria-stabilized zirconia (YSZ) is a representative solid electrolyte used in SOFCs, and its ionic conductivity has been well investigated as a function of dopant concentration, and 8 mol % YSZ (8YSZ) exhibits the highest ionic conductivity.^7^ In bulk 8YSZ, the dopants are randomly distributed, and the effect of the configuration on ionic conductivity is generally negligible. In contrast, thin film 8YSZ exhibits a wide range of ionic conductivity,^8^ suggesting a strong dependence on various microscopic structural features, including dopant configuration. Thus, if microscopic structures can be well controlled, the ionic conductivity of 8YSZ could be further enhanced. However, elucidating this microscopic structure–conductivity relationship experimentally remains challenging, motivating computational approaches to investigate it. Recently, we used kinetic Monte Carlo (KMC) simulations^9–11^ to investigate drift currents arising from anisotropic oxygen-ion diffusion under an applied bias voltage while varying the dopant configuration in bulk 8YSZ, thereby demonstrating that dopant configurations have a profound effect on ionic conductivity. Furthermore, we established a link between microscopic dopant configurations and ionic conductivities using a structural descriptor related to ion-migration paths.^11^ In other words, ionic conductivity was explicitly shown to depend systematically on the microscopic dopant configuration.

The ultimate goal is not solely to establish a link between dopant configurations and ionic conductivity, but to identify the configuration that maximizes ionic conductivity based on the link. This is the essence of atomistic inverse design.^12–14^ However, since KMC only solves the forward problem of predicting ionic conductivity from a given configuration, achieving inverse design requires efficient exploration of the vast configuration space. To this end, black-box optimization (BBO)^15^ is particularly well studied. BBO uses a surrogate model constructed from a small configuration-conductivity data set to iteratively propose and evaluate configurations, thereby identifying configurations with the desired ionic conductivity without requiring knowledge of the full configuration–conductivity relationship. That said, identifying optimal atomic configurations in large multicomponent crystalline systems is challenging due to the combinatorial explosion in the number of possible configurations, which renders conventional BBO methods such as Bayesian optimization^16,17^ intractable. In such situations, Factorization Machine with Quadratic-optimization Annealing (FMQA)^18,19^ provides a powerful alternative, combining the efficiency of BBO with the combinatorial search capability of Ising machines.^20^ Recently, we applied FMQA with Vienna Ab initio Simulation Package (VASP) to determine stable atomic configurations in a small supercell^21^ and in a relatively large grain boundary system,^22^ thereby elucidating their atomic configurations. FMQA has thus emerged as a key technology in BBO, with successful applications ranging from crystal structure prediction to broader combinatorial configuration problems.^23–26^

Here, we demonstrate that the atomistic inverse design problem for solid electrolytes can be solved by combining KMC^9–11^ with FMQA,^18,19^ establishing an efficient structural design framework (KMC–FMQA framework), as illustrated in Figure 1. KMC evaluates the ionic current for a given dopant configuration, and FMQA iteratively learns a surrogate model from a small configuration-current data set and proposes configurations predicted to exhibit the desired ionic current. We apply this framework to optimize the dopant configuration in a 16-layer region of a bulk 8YSZ supercell (see Figure 1(c)). For efficient exploration of the vast configuration space, we introduce a block model in which four lattice sites are grouped into a single unit (block), and three types of blocks, Zr_4_, Y_2_Zr_2_, and Y_4_, are used to construct the 8YSZ supercell (see Figure 1(a)). To maintain the 8YSZ dopant concentration, we enumerated all 81 possible block compositions (*b* = 0–80) that 320 Y atoms were included in 200 blocks within the 16 layers, as shown in Figure 2(a) (see Supporting Note B for details). We then partitioned the search space into 81 compositional subspaces and explored each subspace independently. In this letter, we set the inverse-design target to identify the block configuration exhibiting the highest ionic conductivity. Further details are described in Methods.

**Figure 1:**
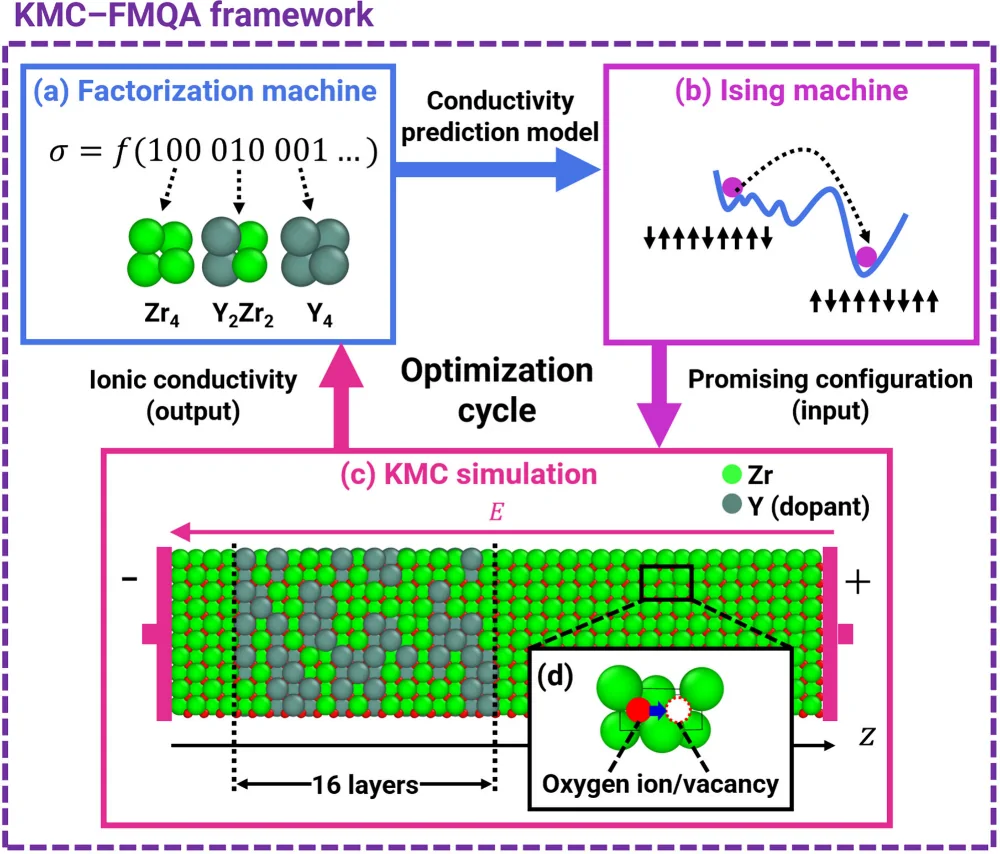
KMC–FMQA framework. In (a), the factorization machine (FM) is trained using the relationship between the dopant configurations encoded as bit strings and their ionic conductivities, and the trained FM is used by an Ising machine (b) to explore promising dopant configurations. In (c), KMC simulations are performed on the systems corresponding to the proposed bit configurations, and the resulting ionic conductivities are added to the training data set for the next cycle. Green and gray spheres represent zirconium (Zr) and yttrium (Y) lattice ions to be optimized, respectively, whereas red spheres represent oxygen ions migrating via oxygen vacancies along paths consisting of six lattice sites (d).

**Figure 2:**
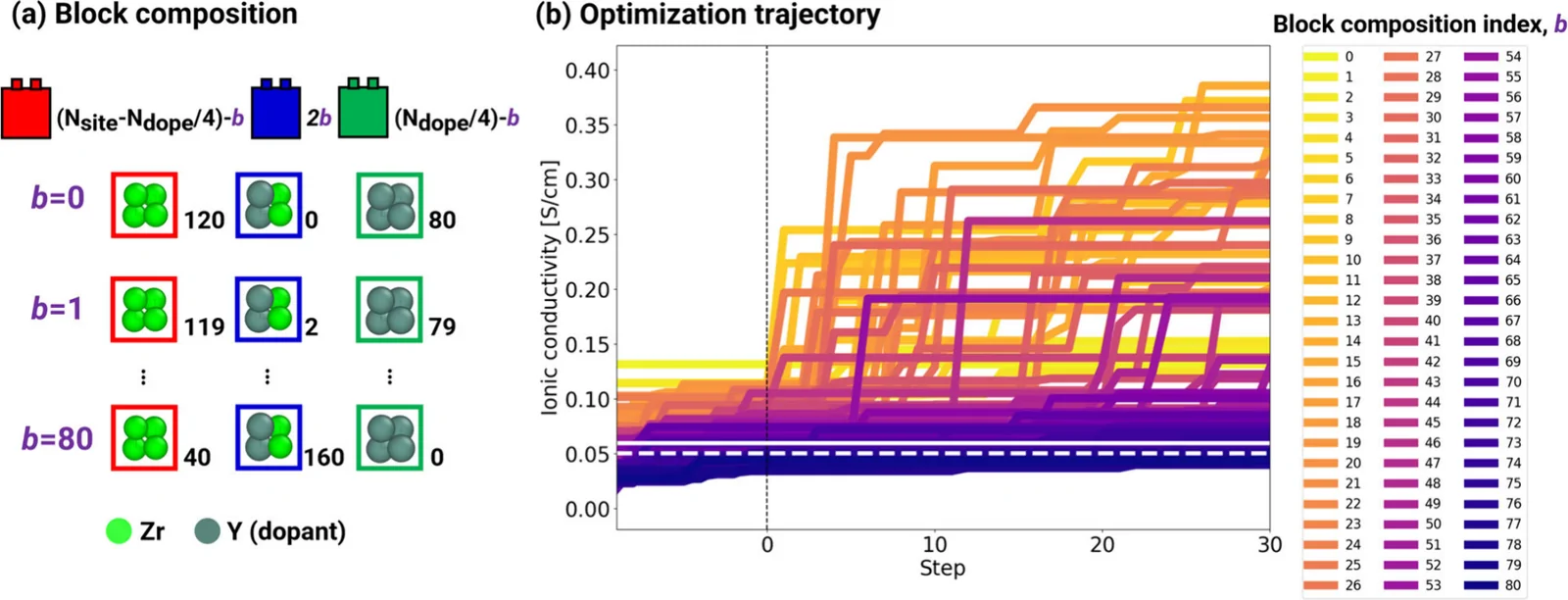
(a) Eighty-one block compositions satisfying the 8YSZ dopant concentration, where *N*_site_ and *N*_dope_ denote the numbers of block placement sites and yttrium (Y) dopants in the system, respectively (*N*_site_ = 200, *N*_dope_ = 320). *b* denotes the block composition index. (b) Optimization trajectories for each of the 81 block compositions. Each trajectory shows the highest ionic conductivity achieved at each iteration step. The ionic conductivity up to step 0 corresponds to the initial training data obtained from 10 evaluations through random sampling, and the subsequent values are obtained through FMQA optimization. The white solid and white dashed horizontal lines represent the average conductivity for random dopant distributions^11^ and the experimentally reported conductivity of bulk 8YSZ,^27^ respectively.

Figure 2(b) shows the evolution of the highest ionic conductivity obtained in 81 parallel KMC–FMQA optimization runs over 30 steps with 10 initial training data. FMQA consistently identified dopant configurations with higher ionic conductivity than those obtained by the initial random sampling, demonstrating the effectiveness of the KMC-FMQA framework. Note that histograms of the ionic conductivities are shown in Figure S1. To further explore promising configurations, an additional 70 optimization steps were performed for the three block compositions with *b* = 20, 14, and 8, that exhibited the highest ionic conductivities during the first 30 steps (see Figure S2). As shown in Figure S2, the optimization trajectories appear to vary significantly depending on the initial data distribution, suggesting that there is room for improving the BBO algorithm to ensure more robust optimization (see Supporting Note E and F).

The optimization identified a dopant configuration with an ionic conductivity of 0.423 S cm^–1^, which is an order-of-magnitude higher than the average conductivity for random dopant distributions (0.06 S cm^–1^).^11^ Note that the calculated conductivity for random dopant distributions is close to the experimentally reported conductivity of bulk 8YSZ (0.0505 S cm^–1^).^27^ Figures 3(a)-(c) show the three highest-conductivity configurations for the three block compositions with *b* = 20, 14, and 8, whereas Figures 3(d)-(f) show the configurations with ionic conductivity close to the experimentally reported value, selected from the initial random sampling data sets for the same block compositions. Note that we searched for high-conductivity dopant configurations in the bulk 8YSZ system in which the dopants are confined to the 16 layers, that is, the investigated system corresponds to the ZrO_2_/YSZ/ZrO_2_ stripe model which has two interfaces in the simulation cell. In the high-conductivity configurations, dopants are highly concentrated near the edges of the 16 layers (i.e., at the YSZ/ZrO_2_ interfaces); the importance of the interfacial structure was also confirmed by the Random Forest regression analysis (see Supporting Note G). Such layered dopant configurations are expected to be fabricated using epitaxial growth techniques such as pulsed laser deposition (PLD) and atomic layer deposition (ALD). The ease of ion migration is determined by the dopant configuration in the six-site ion-migration path shown in Figure 1(d) and by the electric field (see our previous study^11^ for details). When dopants are locally concentrated in the path, the configuration-dependent migration barrier tends to be higher (see Table S2). We thus investigated the electrostatic contribution to the activation barrier around the layered dopant configurations (see Supporting Note H), but a definitive explanation for the observed enhancement in conductivity remains elusive. To fully elucidate the configuration–conductivity relationship, further investigations are required, including exploring a broader range of dopant configurations and identifying key structural features from high-dimensional structural descriptors governing ionic conductivity.^11^

**Figure 3:**
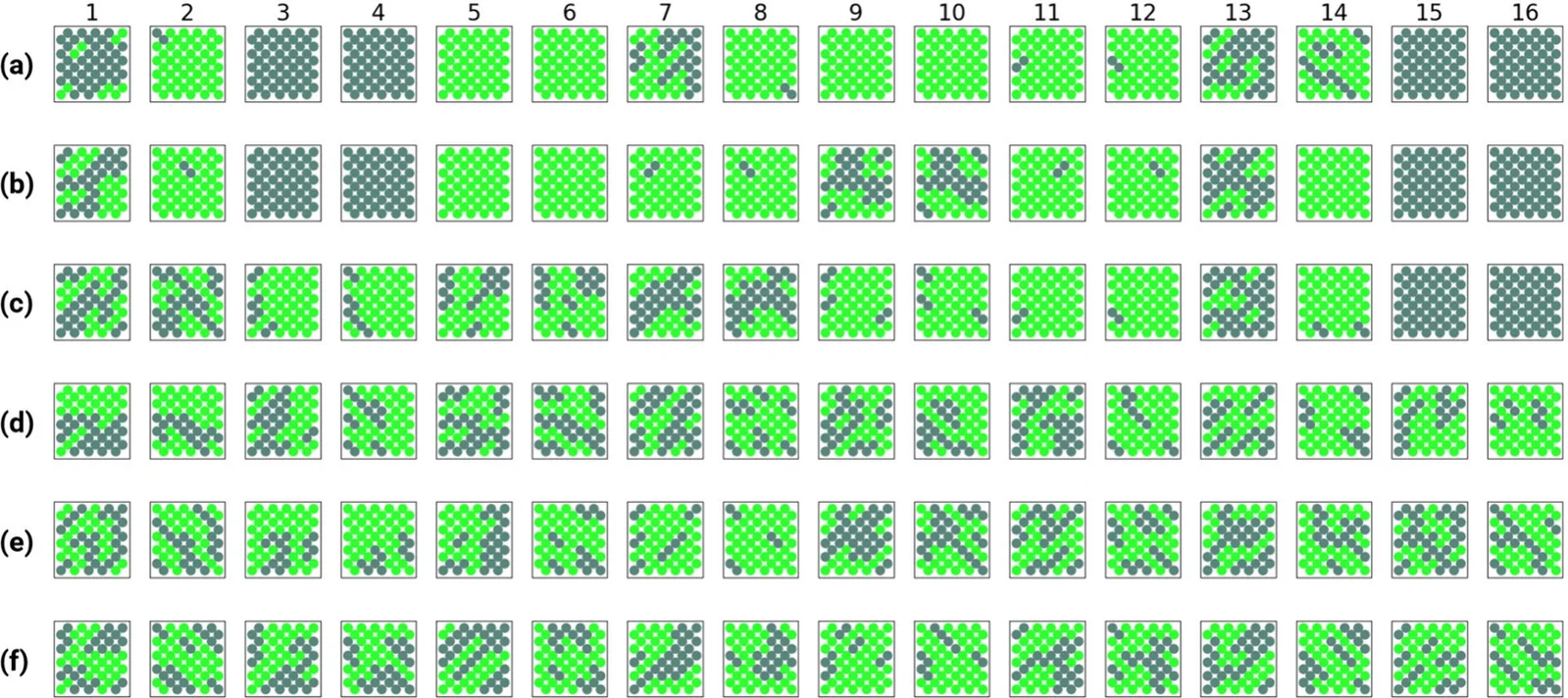
Cross-sectional images of high-conductivity configurations sliced into 16 layers. (a)-(c) The three highest-conductivity configurations identified by the KMC-FMQA framework, with ionic conductivities of 0.423, 0.400, and 0.371 S cm^–1^, respectively. (d)-(f) Configurations with ionic conductivity close to the experimentally reported value, selected from the initial random data sets corresponding to (a)-(c). Green and gray spheres represent zirconium (Zr) and yttrium (Y) atoms, respectively.

To benchmark the framework, we compared three approaches with progressively reduced search spaces: the all-atom model, the block model without the composition-based partitioning based on the block composition index *b*, and the block model with the partitioning (see Supporting Note A). The block model with composition-based partitioning achieved the best optimization performance among the three approaches, demonstrating that a block-based representation combined with search-space partitioning is more effective than simply increasing the number of trials in a vast search space.

In summary, we developed KMC–FMQA framework and applied it to search for high-conductivity dopant configurations in the bulk 8YSZ system. As a proof of concept, we demonstrated that the framework enables the atomistic inverse design of solid electrolytes, identifying a dopant configuration with an order-of-magnitude higher ionic conductivity than that of random configurations. The framework efficiently explores the vast configuration space through a block-based representation combined with composition-based partitioning, enabling rapid identification of high-conductivity configurations. To ensure more practical exploration, we plan to take energetic stability into account in the framework. In the future, by minimizing the discrepancy between experimental and calculated conductivities, the framework will enable inverse estimation of microscopic structures, potentially providing deeper microscopic insight into ionic transport mechanisms in SOFC electrolytes. The approach is readily extensible to electrolyte-electrode interfaces, opening a pathway toward broader atomistic materials design for SOFC performance optimization.

## Methods

An overview of the KMC–FMQA framework is summarized in Figure 1. KMC evaluates the ionic conductivity for a given dopant configuration, whereas FMQA learns a surrogate model from a small configuration-conductivity data set and optimizes the dopant configuration to maximize ionic conductivity. Here, we first briefly explain the KMC evaluation and then describe the FMQA optimization.

In KMC part, for given dopant configurations, KMC evaluates drift currents arising from the migration of mobile light oxygen ions (Figure 1(d)), where the migration barriers mainly depend on the dopant configuration (see Table S2 for the barrier parameters). In contrast, FMQA updates dopant configurations to achieve the desired current. That is, KMC is employed to simulate the time evolution of light mobile-ions, whereas FMQA updates the configuration of heavy lattice-atoms. When the optimization targets energetic stability rather than ionic conductivity, the roles of KMC and FMQA become analogous to those of MD and MC in MC–MD algorithms,^28^ respectively. The KMC computational conditions are summarized in Table S1 and are described in detail in our previous study.^11^ In the previous study, we observed diverse ionic conductivities when dopants were highly concentrated in 16 layers along an applied bias voltage direction shown in Figure 1(c). Therefore, in this study, the KMC–FMQA framework was applied to the optimization of dopant configurations in the 16 layers between the dashed lines in Figure 1(c).

To efficiently handle the large configuration space, we introduced a block model based on the six-site ion-migration paths that govern ionic conductivity^11^ (see Supporting Note B for details), even though this reduction of the search space may not be appropriate for the inverse estimation of microscopic structures when combined with experiments. This model significantly reduced the search space, yielding a Quadratic Unconstrained Binary Optimization (QUBO) model that can be efficiently solved using an Ising machine. The 16 layers were constructed using three types of blocks (see Figure 1(a)), and 81 block compositions satisfying the 8YSZ dopant concentration were enumerated in advance (see Figure 2(a)). The search space reduction is provided in Supporting Note C.

FMQA was employed to optimize the block configuration to maximize ionic conductivity. Since only binary variables (0/1) can be used as inputs for FMQA, the block configurations were represented as bit structures; the three blocks were represented as 100, 010, and 001, as illustrated in Figure 1(a). The bit *q*_*it*_ satisfies the following two constraints

1 $$\overset{3}{\underset{t=1}{\sum }}q_{it}=1,\forall i$$

where *i* and *t* denote the site and block-type indices, respectively, and when constructing the system by a block composition with index *b*,

2 $$\overset{N_{site}}{\underset{i=1}{\sum }}q_{it}=N_{t}\left(b\right)$$

where *N*_*t*_(*b*) denotes the number of blocks of type *t*, and *N*_site_ denotes the number of block placement sites. The first constraint ensures that each site is assigned exactly one block type (one-hot constraint), and the second fixes the block composition to satisfy the 8YSZ dopant concentration. The optimization was performed as following four steps.

1. Initial Training Data Generation: 10 block configurations were generated by random sampling under the two constraints of eqs 1 and 2, and KMC simulations were performed to obtain the corresponding ionic conductivities.
2. Model Training: The factorization machine (FM) was trained using the initial data set. The ionic conductivity prediction model is defined as follows.

3 $$\sigma \left(q\right)=\overset{N_{site}}{\underset{i=1}{\sum }}\overset{3}{\underset{t=1}{\sum }}w_{it}^{*}q_{it}+\overset{N_{site}}{\underset{i=1}{\sum }}\overset{3}{\underset{t=1}{\sum }}\overset{N_{site}}{\underset{j=1}{\sum }}\overset{3}{\underset{u=1}{\sum }}\overset{K}{\underset{k=1}{\sum }}v_{itk}^{*}v_{juk}^{*}q_{it}q_{ju}$$

where **q** denotes a block configuration represented as a bit structure, *w*_it_^*^ and *v*_itk_^*^ denote the trained parameters, and *K* denotes the dimension of the latent space (hyperparameter) which was set to 8 (default value).^29^

3. Ising Machine Optimization: Ising machine searches for the global maximum for a given function. In this study, Fixstars Amplify AE,^30^ a classical annealer, was employed as the Ising machine and performed Simulated Annealing. A promising configuration **q** that maximizes the trained FM is determined by an Ising machine under the two constraints of eqs 1 and 2. The most promising solution, q*, was selected in each cycle. If q* violated the constraints or was already included in the training data, q* was regenerated by random sampling.
4. Ionic Conductivity Calculation: The KMC simulation is performed on the system identified by q*. The obtained conductivity is added to the training data, and the procedure then returns to Step 2.

The crystalline system exhibits high symmetry; however, the physically equivalent configurations are treated as distinct points in the bit structure space. Therefore, to cover all symmetry-equivalent configurations with a single evaluation, 100 symmetry operations were applied (see Supporting Note D), thereby expanding the training data set by a factor of 100 from a single KMC run. In this study, for each of the 81 block compositions, we performed three independent optimization trials with different initial training data sets. We then determined the highest conductivity in three independent trials and plotted it as a function of optimization step in Figure 2(b).

## Supplementary Material



## Notes

KMC condition; Migration barrier table; Comparison of ionic conductivity histograms from random sampling and FMQA; Optimization trajectories with three highest ionic conductivities; Optimization performance under different constraints; Block model; Search space reduction; Symmetry operations; Analysis of BBO performance; Prediction accuracy of FM; Analysis of structure-conductivity relationship; Electrostatic contribution (PDF)

## Abbreviations

Factorization machine with quadratic-optimization annealing: FMQA
Quadratic unconstrained binary optimization: QUBO
Kinetic Monte Carlo: KMC
Eight mol % yttria-stabilized zirconia: 8YSZ
Solid oxide fuel cells: SOFCs
