# Supplementary material for: Kinetic Monte Carlo–Ising Machine Optimization for Atomistic Inverse Design of Solid Electrolytes
Source: J Phys Chem Lett. 2026 Sep 3;17(37):10758–62. doi: 10.1021/acs.jpclett.6c02157 (PMC13592075; doi:10.1021/acs.jpclett.6c02157)
Supplement: Supplementary file 1 [file jz6c02157_si_001.pdf]

Supporting Information for "Kinetic Monte Carlo–Ising Machine Optimization for Atomistic Inverse Design of Solid Electrolytes"

Ai Koizumi[1,2]*, Tomofumi Tada[3]*, Ryo Tamura[1,4]*

1 Center for Basic Research on Materials, National Institute for Materials Science, 1-1 Namiki, Tsukuba, Ibaraki 305-0044, Japan

2 Center for Computational Sciences, University of Tsukuba, 1-1-1 Tennodai, Tsukuba, Ibaraki 305-8577, Japan

3 Kyushu University Platform of Inter/Transdisciplinary Energy Research, Kyushu University, 744 Motooka, Nishi-ku, Fukuoka 819-0395, Japan

4 Graduate School of Frontier Sciences, The University of Tokyo, 5-1-5 Kashiwa-no-ha, Kashiwa, Chiba 277-8561, Japan

Email: koizumi.ai@ccs.tsukuba.ac.jp, tada.tomofumi.054@m.kyushu-u.ac.jp, TAMURA.Ryo@nims.go.jp.

Table S1 *KMC Condition for calculating ionic conductivity.* See Table S3 in our previous study[1] for other detailed simulation conditions.

Temperature	1050 K
Number of equilibration steps	1000
Number of sampling steps	8000000
Dielectric constant	27
Bias voltage	0.0002 V/Å

Table S2 *Oxygen migration energy barriers* $E^a(A,B,C,D,E,F)$. The 6-site migration path is defined in Figure S4(a). The migration energy barriers with indices up to 42 were calculated by Pornprasertsuk *et al.*[2], whereas those with indices greater than 42 were estimated in our previous study[1] following the method of Grieshammer *et al.*[3] Reproduced from Ref. 1. https://pubs.acs.org/jpccck/article/130/10/3897/5088689/Linking-Microscopic-Structures-to-Ionic-Currents

Index	A	B	C	D	E	F	E^a [eV]	Index	A	B	C	D	E	F	E^a [eV]
1	Zr	Zr	Zr	Zr	Zr	Zr	0.67	33	Zr	Y	Y	Y	Zr	Zr	1.2
2	Y	Zr	Zr	Zr	Zr	Zr	0.2	34	Zr	Y	Y	Zr	Y	Zr	1.09
3	Zr	Y	Zr	Zr	Zr	Zr	0.2	35	Zr	Y	Y	Zr	Zr	Y	0.79
4	Zr	Zr	Y	Zr	Zr	Zr	1.19	36	Zr	Y	Zr	Y	Y	Zr	1.09
5	Zr	Zr	Zr	Y	Zr	Zr	1.19	37	Zr	Y	Zr	Y	Zr	Y	0.79
6	Zr	Zr	Zr	Zr	Y	Zr	0.8	38	Zr	Y	Zr	Zr	Y	Y	0.7
7	Zr	Zr	Zr	Zr	Zr	Y	0.8	39	Zr	Zr	Y	Y	Y	Zr	1.4
8	Y	Y	Zr	Zr	Zr	Zr	0.23	40	Zr	Zr	Y	Y	Zr	Y	1.4
9	Y	Zr	Y	Zr	Zr	Zr	0.88	41	Zr	Zr	Y	Zr	Y	Y	0.94
10	Y	Zr	Zr	Y	Zr	Zr	0.88	42	Zr	Zr	Zr	Y	Y	Y	0.94
11	Y	Zr	Zr	Zr	Y	Zr	0.39	43	Zr	Zr	Y	Y	Y	Y	1.01
12	Y	Zr	Zr	Zr	Zr	Y	0.62	44	Zr	Y	Zr	Y	Y	Y	1.22
13	Zr	Y	Y	Zr	Zr	Zr	0.88	45	Zr	Y	Y	Zr	Y	Y	1.22
14	Zr	Y	Zr	Y	Zr	Zr	0.88	46	Zr	Y	Y	Y	Zr	Y	1.23
15	Zr	Y	Zr	Zr	Y	Zr	0.62	47	Zr	Y	Y	Y	Y	Zr	1.23
16	Zr	Y	Zr	Zr	Zr	Y	0.39	48	Y	Zr	Zr	Y	Y	Y	1.22
17	Zr	Zr	Y	Y	Zr	Zr	1.23	49	Y	Zr	Y	Zr	Y	Y	1.22
18	Zr	Zr	Y	Zr	Y	Zr	0.71	50	Y	Zr	Y	Y	Zr	Y	1.23
19	Zr	Zr	Y	Zr	Zr	Y	0.71	51	Y	Zr	Y	Y	Y	Zr	1.23
20	Zr	Zr	Zr	Y	Y	Zr	0.71	52	Y	Y	Zr	Zr	Y	Y	0.67
21	Zr	Zr	Zr	Y	Zr	Y	0.71	53	Y	Y	Zr	Y	Zr	Y	0.8
22	Zr	Zr	Zr	Zr	Y	Y	0.45	54	Y	Y	Zr	Y	Y	Zr	0.8
23	Y	Y	Y	Zr	Zr	Zr	0.7	55	Y	Y	Y	Zr	Zr	Y	0.8
24	Y	Y	Zr	Y	Zr	Zr	0.7	56	Y	Y	Y	Zr	Y	Zr	0.8
25	Y	Y	Zr	Zr	Y	Zr	0.28	57	Y	Y	Y	Y	Zr	Zr	0.79
26	Y	Y	Zr	Zr	Zr	Y	0.28	58	Zr	Y	Y	Y	Y	Y	1.26
27	Y	Zr	Y	Y	Zr	Zr	1.2	59	Y	Zr	Y	Y	Y	Y	1.26

28	Y	Zr	Y	Zr	Y	Zr	0.79	60	Y	Y	Zr	Y	Y	Y	1.19
29	Y	Zr	Y	Zr	Zr	Y	1.09	61	Y	Y	Y	Zr	Y	Y	1.19
30	Y	Zr	Zr	Y	Y	Zr	0.79	62	Y	Y	Y	Y	Zr	Y	0.84
31	Y	Zr	Zr	Y	Zr	Y	1.09	63	Y	Y	Y	Y	Y	Zr	0.84
32	Y	Zr	Zr	Zr	Y	Y	0.7	64	Y	Y	Y	Y	Y	Y	1.23

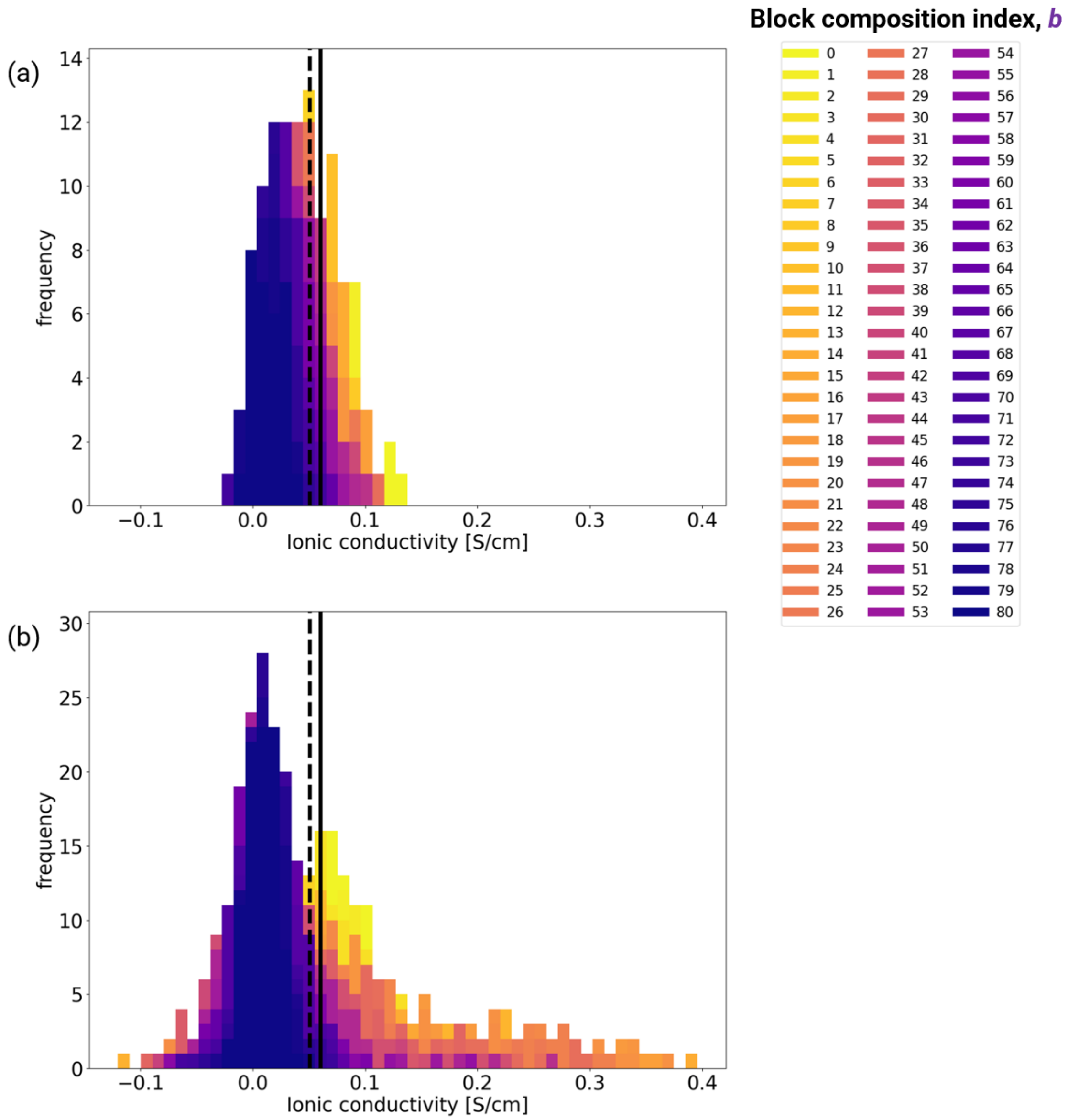

Figure S1 *Comparison of ionic conductivity histograms from random sampling (a) and FMQA (b).* Both panels (a) and (b) show 81 histograms, one for each of the 81 block compositions; the composition index *b* is defined in Fig. S4(e). The 81 histograms in (a) were obtained using the initial training data generated through random sampling (N=10×3 for each block composition), whereas those in (b) were obtained using data generated through Ising optimization (N=30×3 for each block composition). The black solid and black dashed vertical lines represent the average conductivity (N=100) for random dopant distributions across the entire system (0.06 S cm^{-1})[1] and the experimentally reported conductivity of bulk 8YSZ (0.0505 S cm^{-1})[4], respectively.

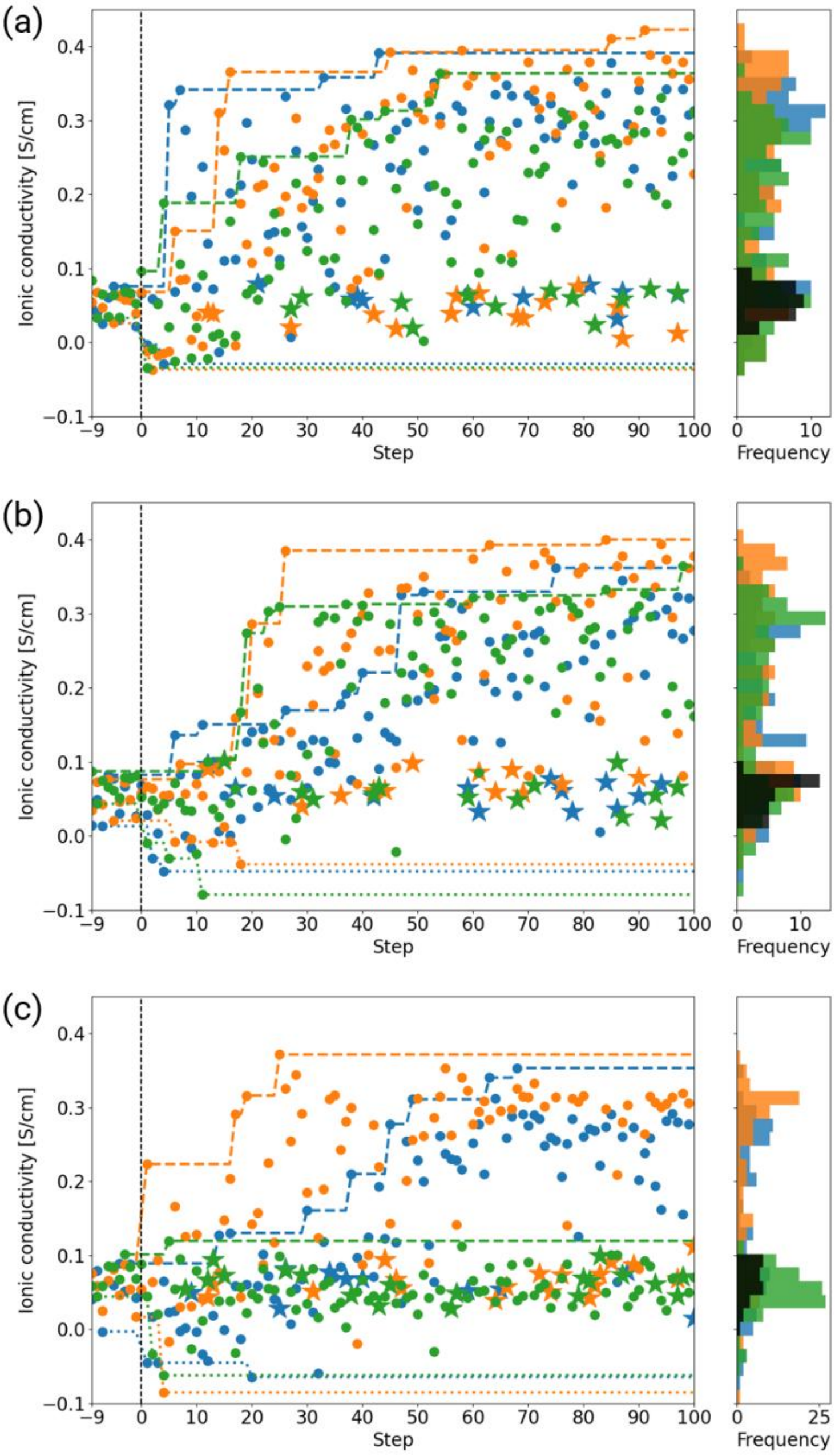

Figure S2 *Three independent optimization trajectories for each of the three block compositions exhibiting the highest conductivities.* Three independent trials are shown in different colors. These three block compositions correspond to b = 20 (a), 14 (b), and 8 (c). Points indicate the ionic conductivities obtained at each step. Star markers represent ionic conductivities of the configurations generated through random sampling when FMQA failed to generate a novel or feasible candidate. The histograms of ionic conductivities for each trial are also plotted on the right side. The black histogram was created using all the initial training data up to the 0th step of the three independent trials.

Supporting Note A: Optimization performance under different constraints

In this study, we evaluated the optimization performance of the block model with block-composition-based partitioning (BWP), discussed in the main text, as well as that of the block model without such partitioning (BWOP) and the all-atom model (AA). In the case of AA, the dopant configuration can be represented using 0 (Zr) and 1 (Y), and the optimization requires only the concentration constraint:

$$\sum_{i}^{N_{\mathrm{site}}} q_i = N_{\mathrm{dope}}. \tag{1}$$

In contrast, for BWOP, the following two constraints, the one-hot constraint and the concentration constraint, are required, as in BWP.

$$\sum_{t}^{3} q_{it} = 1, \forall i, \tag{2}$$

$$\sum_{i}^{N_{\mathrm{site}}} \sum_{t}^{3} N_t^{\mathrm{Y}} q_{it} = N_{\mathrm{dope}}, \tag{3}$$

where N_t^{Y} denotes the number of Y atoms constructing the block of type t. The upper panel of Figure S3 shows the optimization trajectories for the two cases, compared with the 81 parallel explorations (i.e., BWP) in the background in gray, whereas the panels (AA) and (BWOP) of Fig. S3 show the trajectories of three independent trials. Although BWOP has a smaller search space than AA, the Ising optimization did not work for BWOP in any of the three trials. Generally, imposing many constraints makes optimization difficult. In both BWP and BWOP, an additional one-hot constraint is required, which may have contributed to the failure of Ising optimization in BWOP. Nevertheless, the 81 parallel explorations (BWP) exhibit the best performance, thereby enabling the rapid identification of microscopic structures with the desired current.

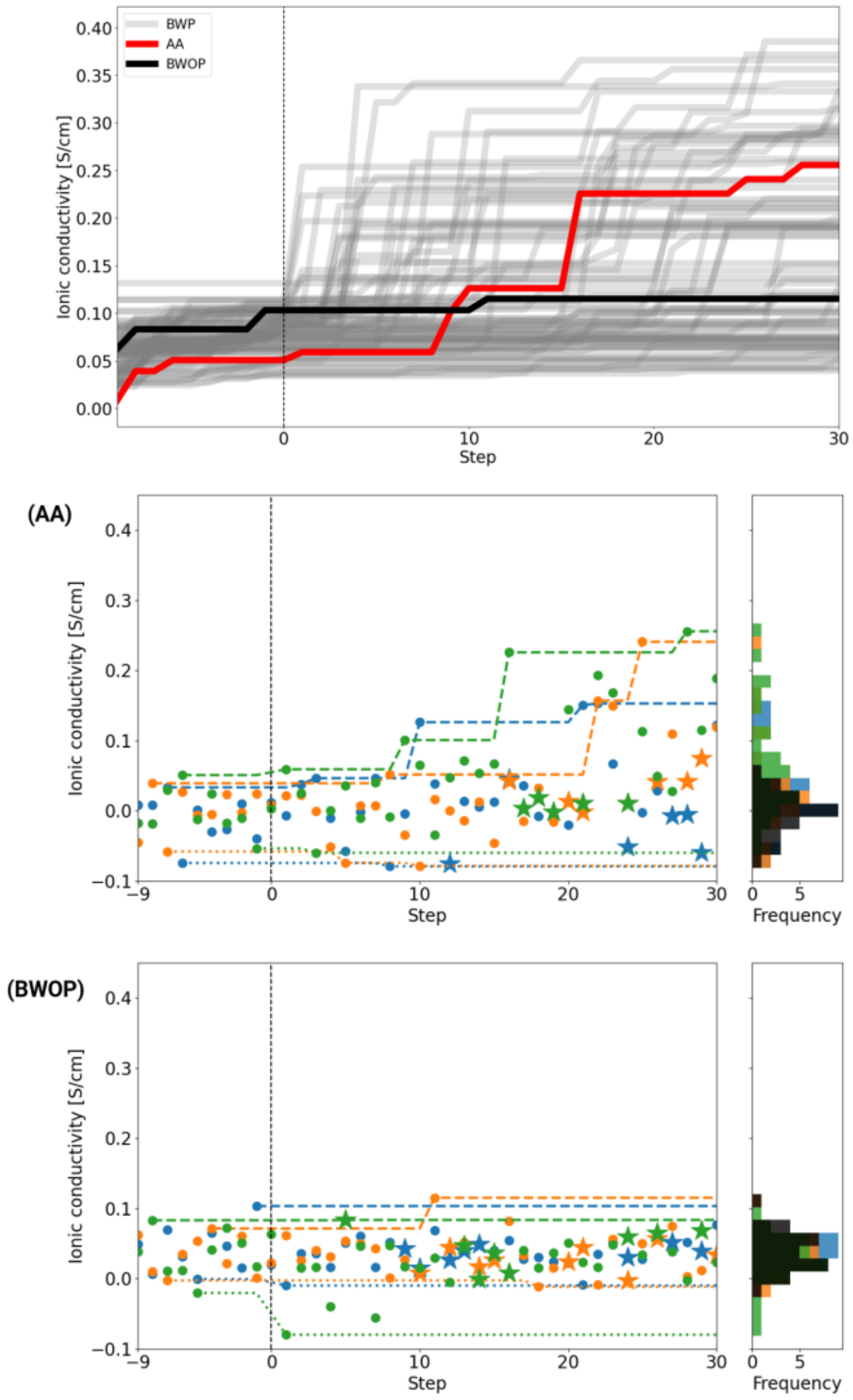

Figure S3 *Optimization trajectories of the two additionally investigated cases.* (Upper panel) Trajectories show the highest ionic conductivity achieved at each iteration step. The highest conductivity of each step was determined in three independent trials with different initial training datasets. The ionic conductivity up to the 0th step corresponds to the initial training data generated through random sampling, whereas the subsequent values are obtained through the Ising optimization. The results of the 81 parallel explorations are also shown in the background in gray for comparison. (Bottom two panels) Optimization trajectories for AA and BWOP. Three independent trials are shown in different colors. Points represent the ionic conductivities obtained at each step. Star markers represent the ionic conductivities of the configurations generated through random sampling when the FMQA failed to generate a novel or a feasible candidate. The histograms of ionic conductivities for each trial are also plotted on the right side. The black histogram was created using all the initial training data from the three independent trials.

Supporting Note B: Block model

We explain the motivation for introducing the block model and the considerations that guided its modeling. In our previous study[1], based on 64 types of six-site ion-migration paths (see Fig. S4(a)), we defined the histograms of the paths that construct the system, and we linked the dopant configurations to ionic conductivity using the histograms. That is, the dopant configurations on the six sites (A–F) along the ion-migration path are important to characterize ionic conductivity. We thus believe that effective control of the ionic conductivity can be achieved by varying the composition and configuration of the 64 paths in the system. To reduce the search space, eight dopant configurations with representative migration energies were selected (see Fig. S4(b)); these configurations exhibit a wide range of migration energies (see the red star markers in Fig. S4(c)). As shown in Fig. S4(d), these eight configurations were decomposed into three types of four-site blocks, Zr_4, Y_2Zr_2, and Y_4. In this study, we used the blocks to construct the 16 layers. To fix the dopant concentration at 8YSZ, we enumerated the 81 block combinations in advance, as shown in Fig. S4(e). For example, the first block composition with b = 0 consists of 120 Zr_4 blocks, 0 Y_2Zr_2 blocks, and 80 Y_4 blocks, and the second with b = 1 consists of 119 Zr_4 blocks, 2 Y_2Zr_2 blocks, and 79 Y_4 blocks, that is, b corresponds to half the number of Y_2Zr_2 blocks in each composition. The optimization was performed for each of 81 block compositions.

Here we briefly discuss the effect of block compositions on ionic conductivity. Figure S5 shows the ionic conductivity as a function of b; each data point represents the average ionic conductivity obtained from initial random sampling (N=10×3) for each block composition. Ionic conductivity decreases as b increases, indicating that the Y_2Zr_2 blocks reduce ionic conductivity. Despite all simulation models being constructed with

the same dopant concentration, we found that the ionic conductivity strongly depends on the block composition.

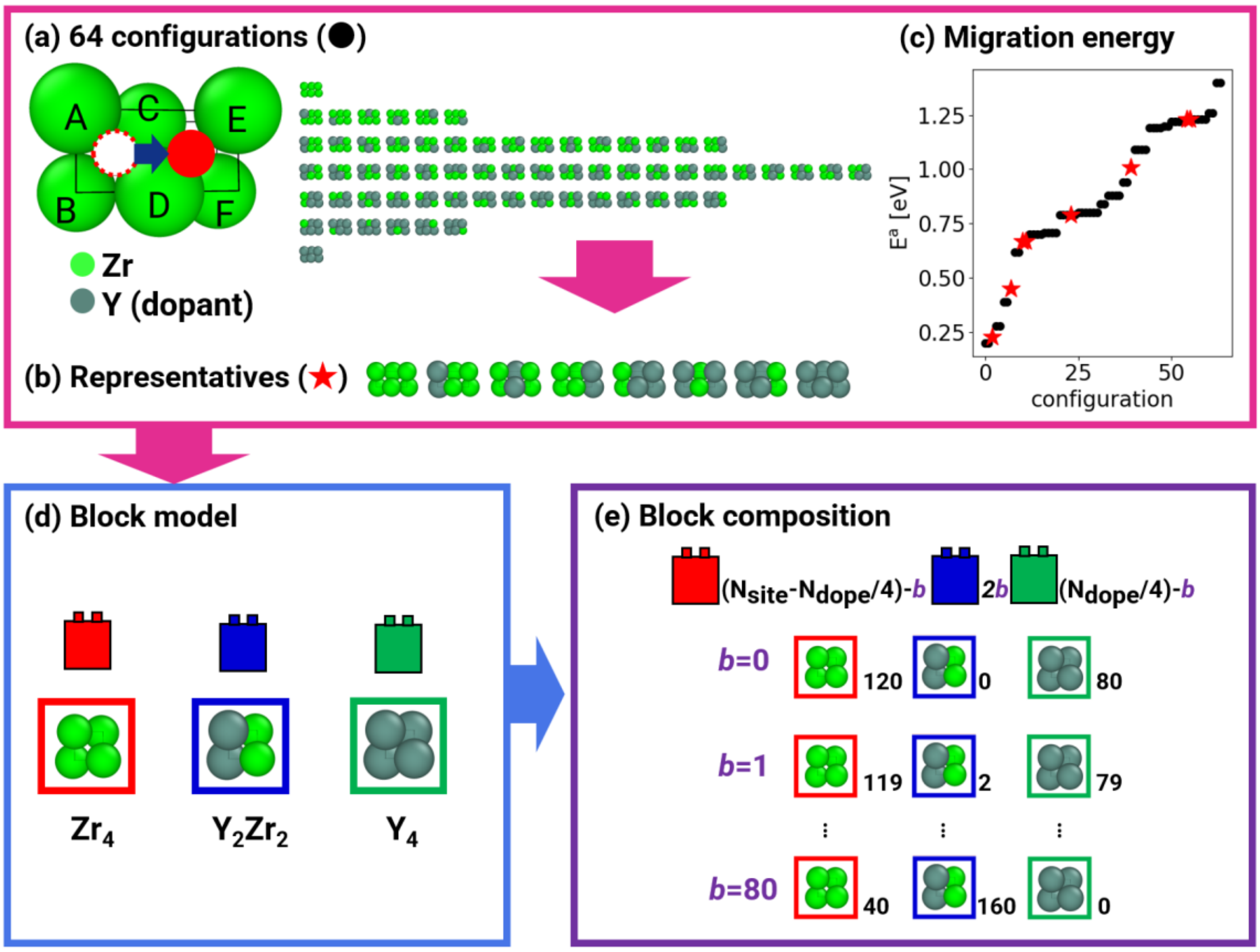

Figure S4 *Schematic illustration of the thought process leading to block model.* (a) All 64 possible dopant configurations on the six sites (A–F) along the ion-migration path in the all-atom model. (b) Eight selected representative configurations. (c) Migration energies of all 64 configurations. The red star markers show the migration energies of the representatives. (d) *Block model*: The representatives were decomposed into three types of four-site blocks. (e) *Block composition*: Eighty-one block combinations satisfying the 8YSZ dopant concentration were used to construct the 16 layers. Green and gray spheres represent zirconium (Zr) and yttrium (Y) atoms, respectively.

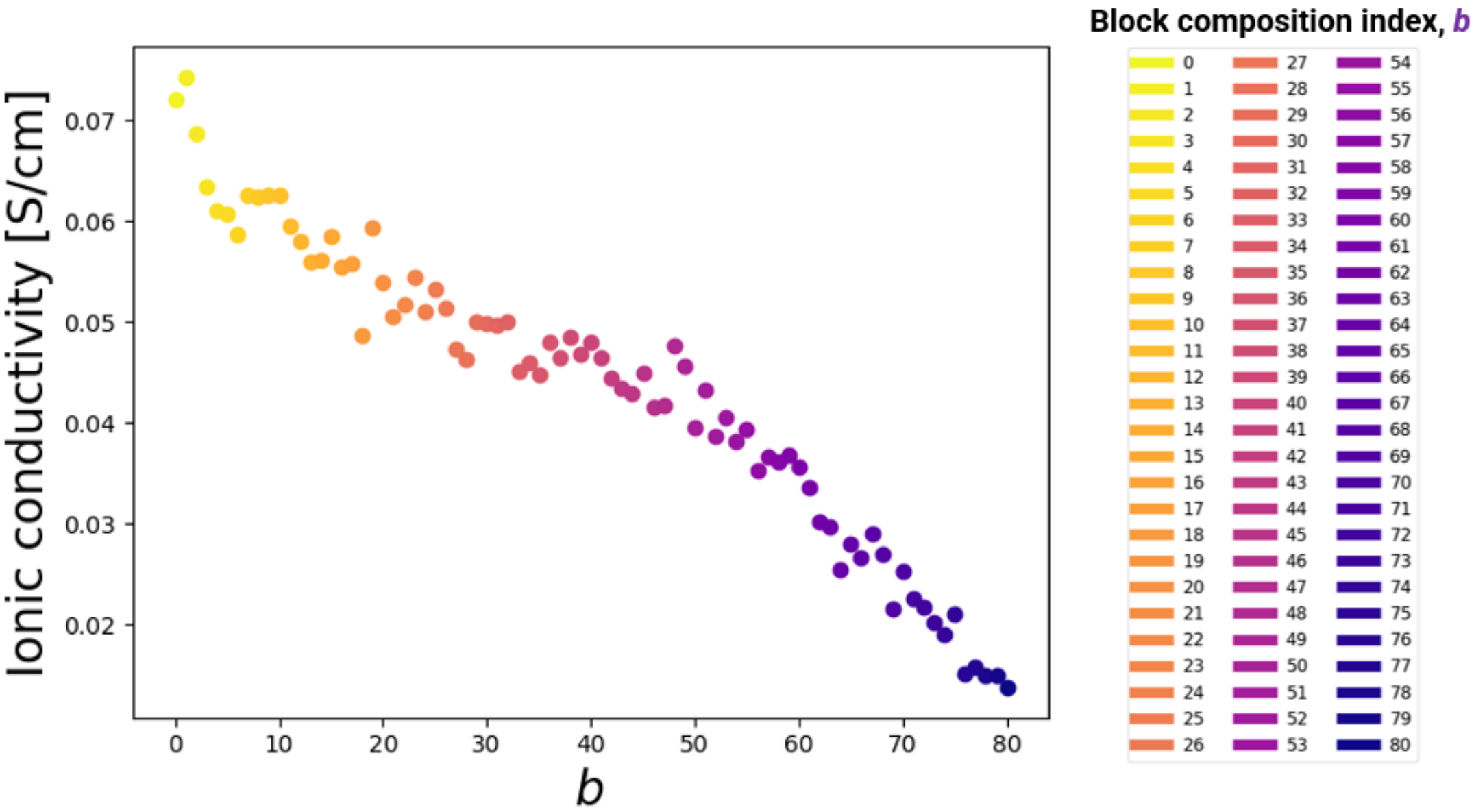

Figure S5 *Ionic conductivity as a function of block composition.*

Supporting Note C: Search space reduction

Here, we describe the size of the search space explored in this study. The size of the search space was reduced by considering the following two points: (i) the dopant configuration in the 16 layers was constructed using three types of four-site blocks, and (ii) the block configuration space was divided into 81 subspaces with different block compositions satisfying the 8YSZ dopant concentration. The search space reduction by considering the block model is summarized in Table S3. The number of possible configurations was reduced to 10^{91} from 10^{232} by considering the block model. The search space with 10^{91} candidates was further divided into 81 subspaces with different block compositions, and the resulting number of possible configurations in each subspace is depicted in Fig. S6.

Table S3 *Number of sites, number of possible configurations, and bit lengths.*

Model	All-Atom	Block
Number of sites, N_{site}	800	200
Unit	Zr/Y	$Zr_4/Y_2Zr_2/Y_4$
Bit length	800	600
Number of possible configurations (logarithmic scale)	232	91

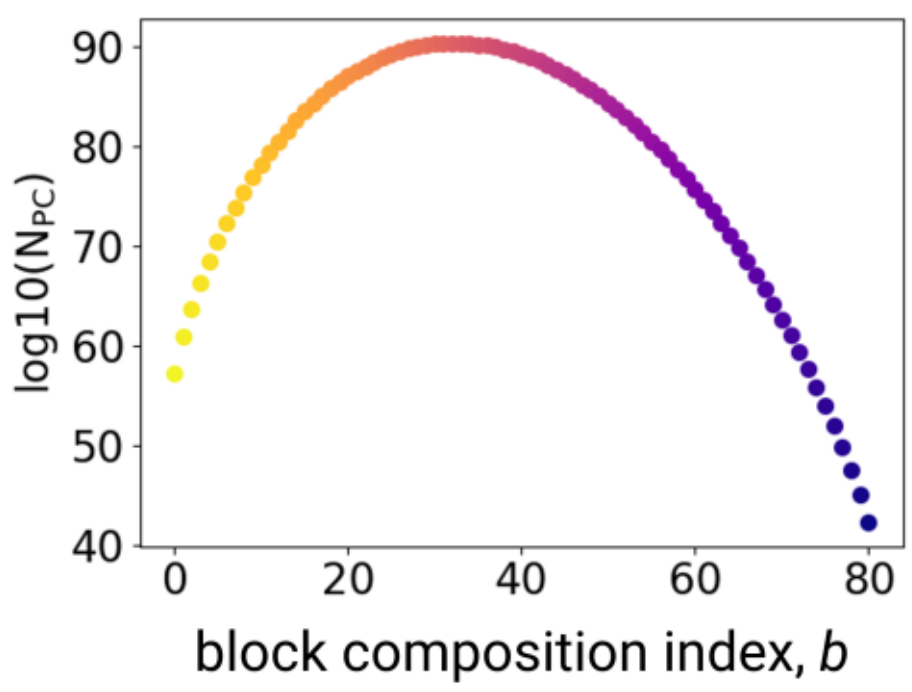

Figure S6 *Number of possible configurations,* N_{PC}*, for each block composition.*

Supporting Note D: Symmetry operations

Here, we describe the symmetry operations considered for the crystalline system, as illustrated in Fig. 1(c). The system has 10 layers in each of the x and y directions, and 40 layers in the z direction. A weak bias voltage was applied in the z direction, and thus we considered symmetry operations for only the x and y directions. The crystalline system is constructed by repeating the four-site block structure, and thus translational operations of 5 × 5 in both the x and y directions are permitted. Additionally, rotation operation around the C_2 axis and mirror operation with respect to the red plane are permitted, as illustrated in Fig. S7(a)-(d). Thus, ultimately, we considered 100 (5 × 5 × 4) symmetry operations in this study.

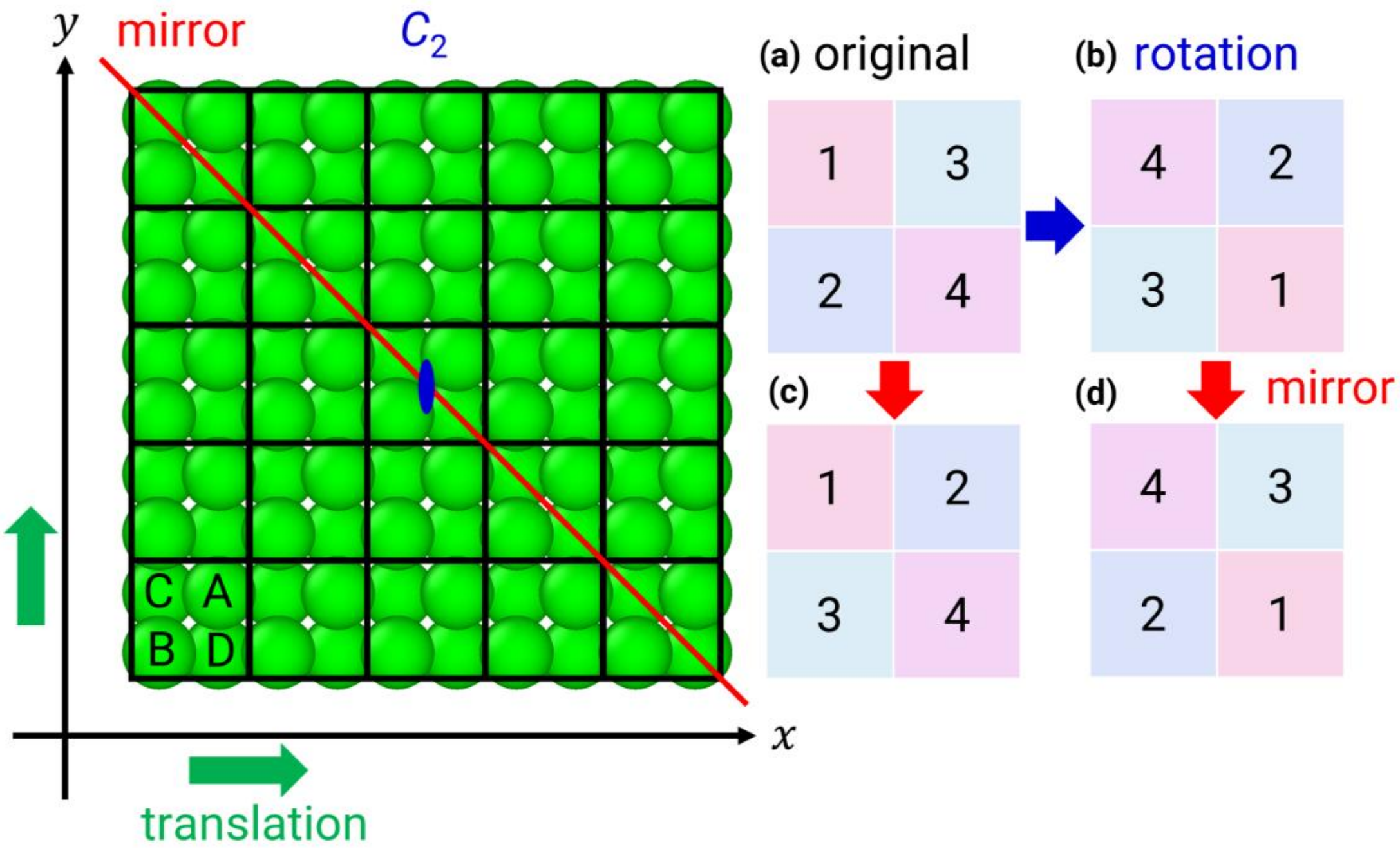

Figure S7 *Symmetry operations.* The crystal structure is shown in the x-y plane, with the z direction perpendicular to the plane of the paper. The red line and blue ellipse indicate the mirror plane and the C_2 axis, respectively, extending perpendicular to the x-y plane (i.e., along the z direction).

Supporting Note E: Analysis of BBO performance

As shown in Figure S2(c), the optimization results appear to vary significantly depending on the initial data distribution. Thus, we investigated the relationship between the BBO performance and the conductivity variance of the initial data to identify the conditions under which BBO performs well. Figure S8 shows the relationship between the conductivity variance of the initial data and the conductivity growth rate over the 30 optimization steps. However, we found no clear relationship.

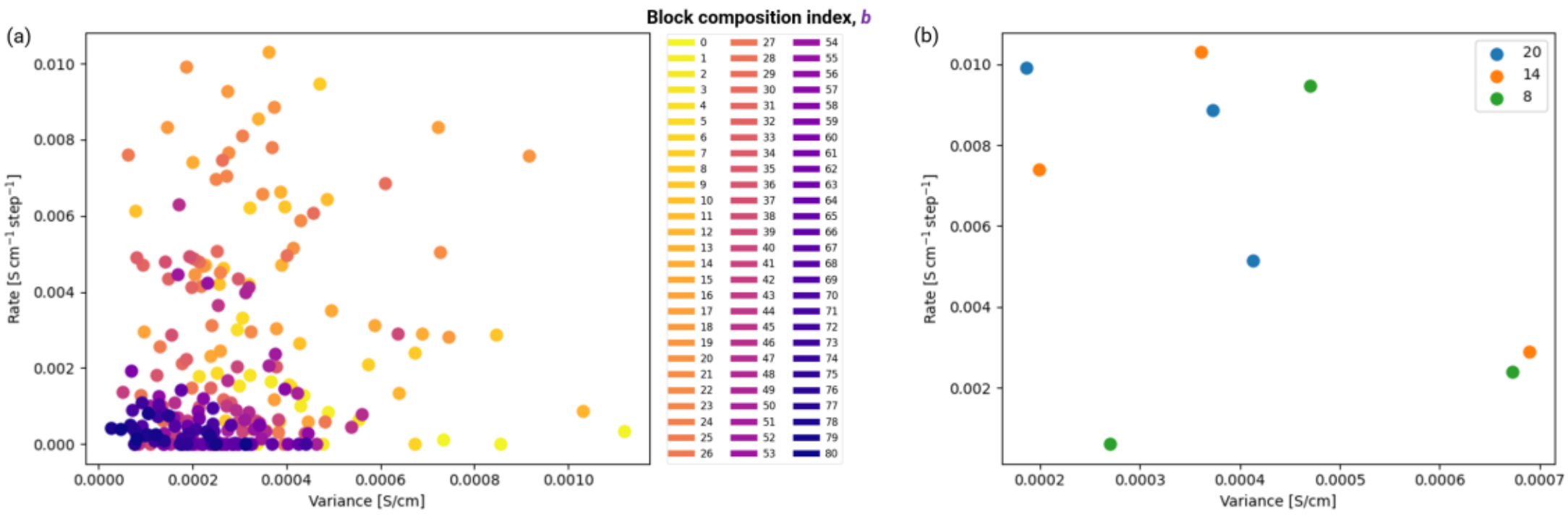

Figure S8 Relationship between the conductivity variance of the initial data and the conductivity growth rate. Panel (a) show the relationships for all block compositions, whereas panel (b) shows those for three block compositions with block composition index b = 20, 14, and 8.

Figure S9 shows the relationship between the block composition index b and the conductivity variance of the initial data, and Figure S10 shows the relationship between the index b and the conductivity growth rate. As shown in Figure S9, we found that the variance of the initial data was large when the index b is small. However, when the index b is small, the growth rate is also very small, as shown in Figure S10. Figure S10 is similar to the relationship between the index b and the number of possible configurations, as shown in Figure S6. Thus, in this study, we conclude that the BBO works well when the

number of possible configurations is large. To understand the BBO behavior, further investigations are needed.

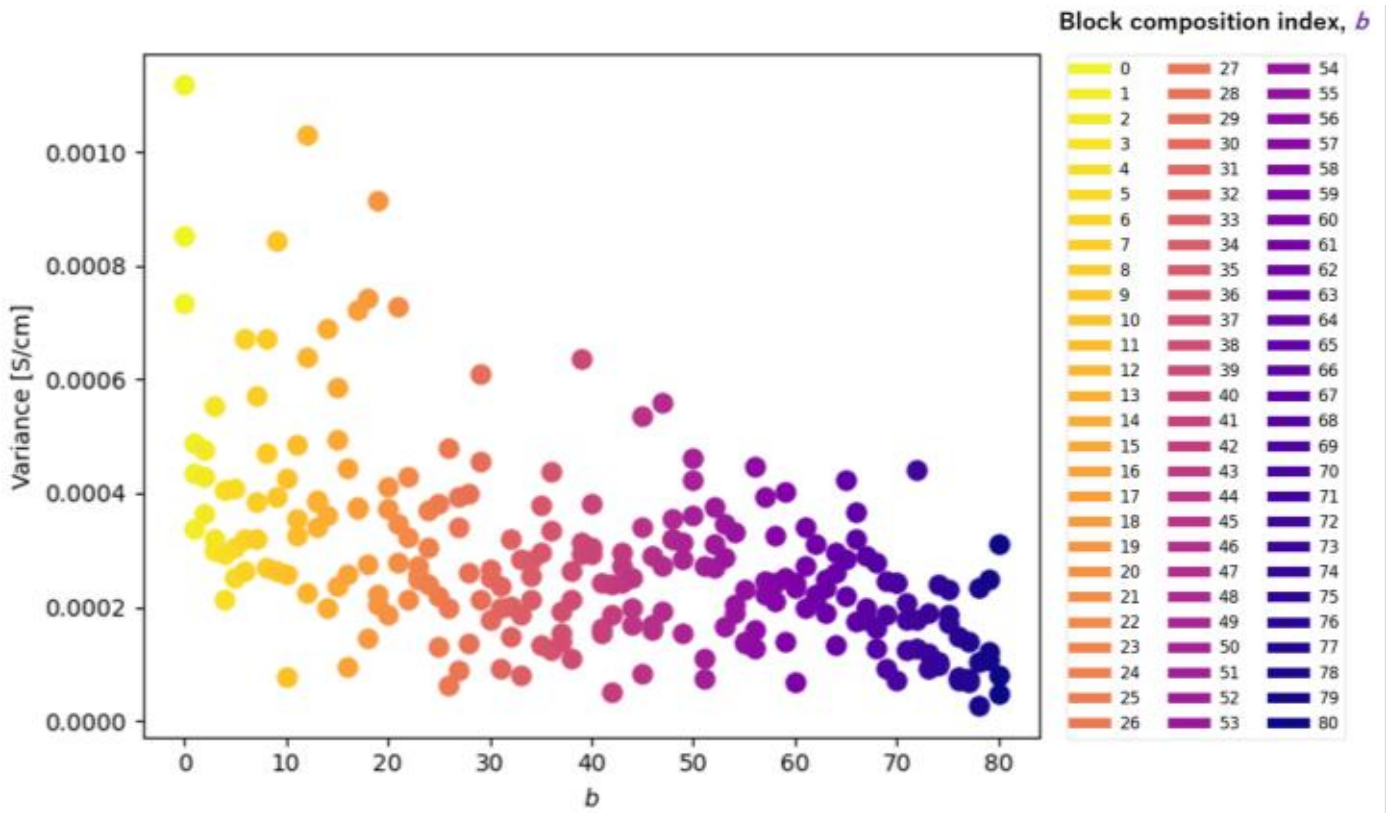

Figure S9 Relationship between the block composition index b and the conductivity variance of the initial data.

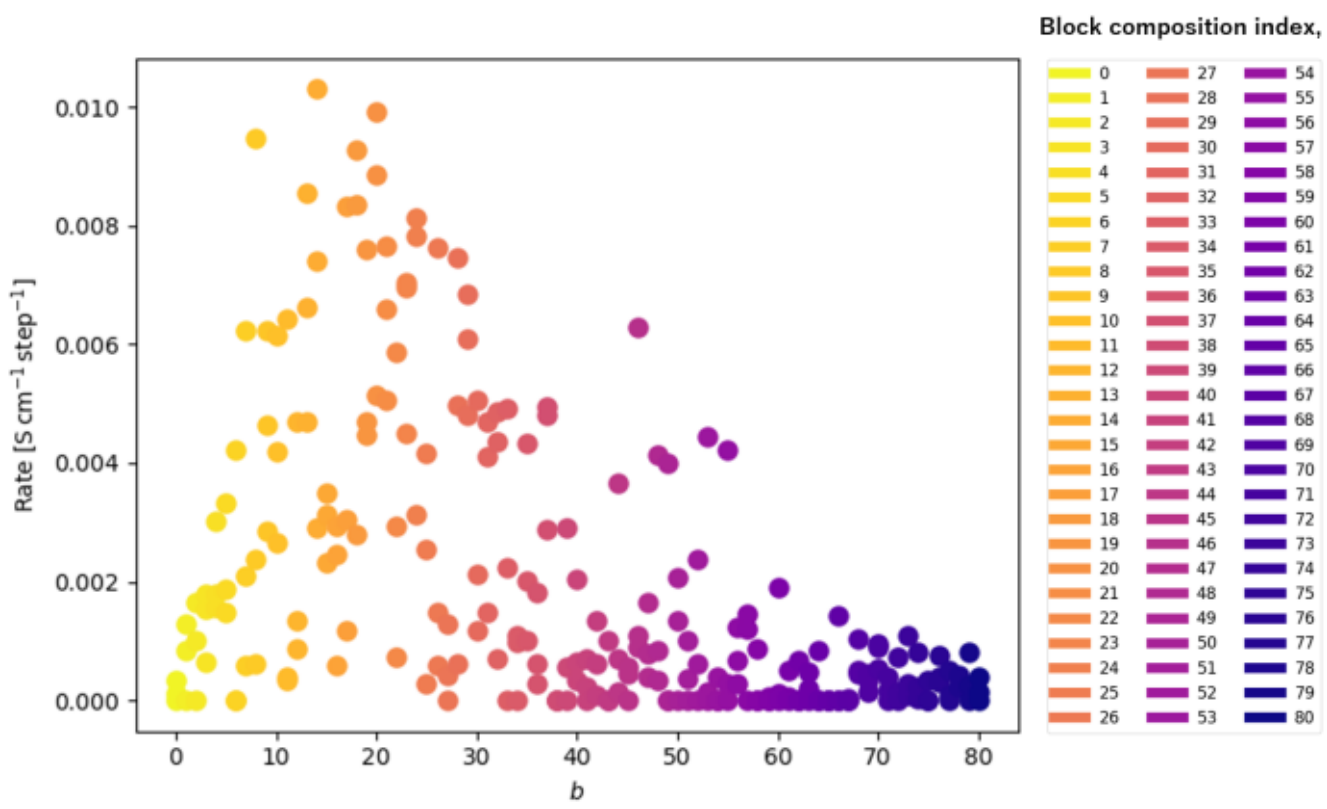

Figure S10 Relationship between the block composition index b and the conductivity growth rate.

Supporting Note F: Prediction accuracy of factorization machine (FM)

We investigated the step dependence of the FM model prediction accuracy for the orange-colored trial shown in Figure S2(a). The upper panels of Figure S11 show scatter plots of the KMC-calculated (Real) versus FM-predicted conductivities for test data using the FM models obtained at five-step intervals from 10 (initial training data) to 40 optimization steps. The test data were obtained from two different trials in Figure S2(a) (N=80). We also investigated two correlation coefficients, Chatterjee's rank-based coefficient[5] and R^2 value, to evaluate the model performance. In Figure S11, Panels (a) and (b) show Chatterjee's coefficient and R^2 value, respectively. As shown in the scatter plots in Figure S11, increasing training data from N=1000 to N=1500 appears to improve the performance of the FM model. In particular, the relative ordering of the conductivities is reproduced more accurately. However, it appears to be worse in the subsequent steps. This tendency is well captured by Chatterjee's coefficient as shown in Figure S11(a), even though the absolute prediction accuracy remains low as shown in Figure S11(b). In the BBO, we believe that the relative ordering prediction is more important than the absolute prediction to search promising candidates. The BBO algorithm is not well suited for exploration, which may lead to biased sampling and degrade the overall performance of the model. Increasing training dataset does not necessarily improve the FM prediction accuracy. Therefore, there is room for improvement in the algorithm, particularly in its exploration capability.

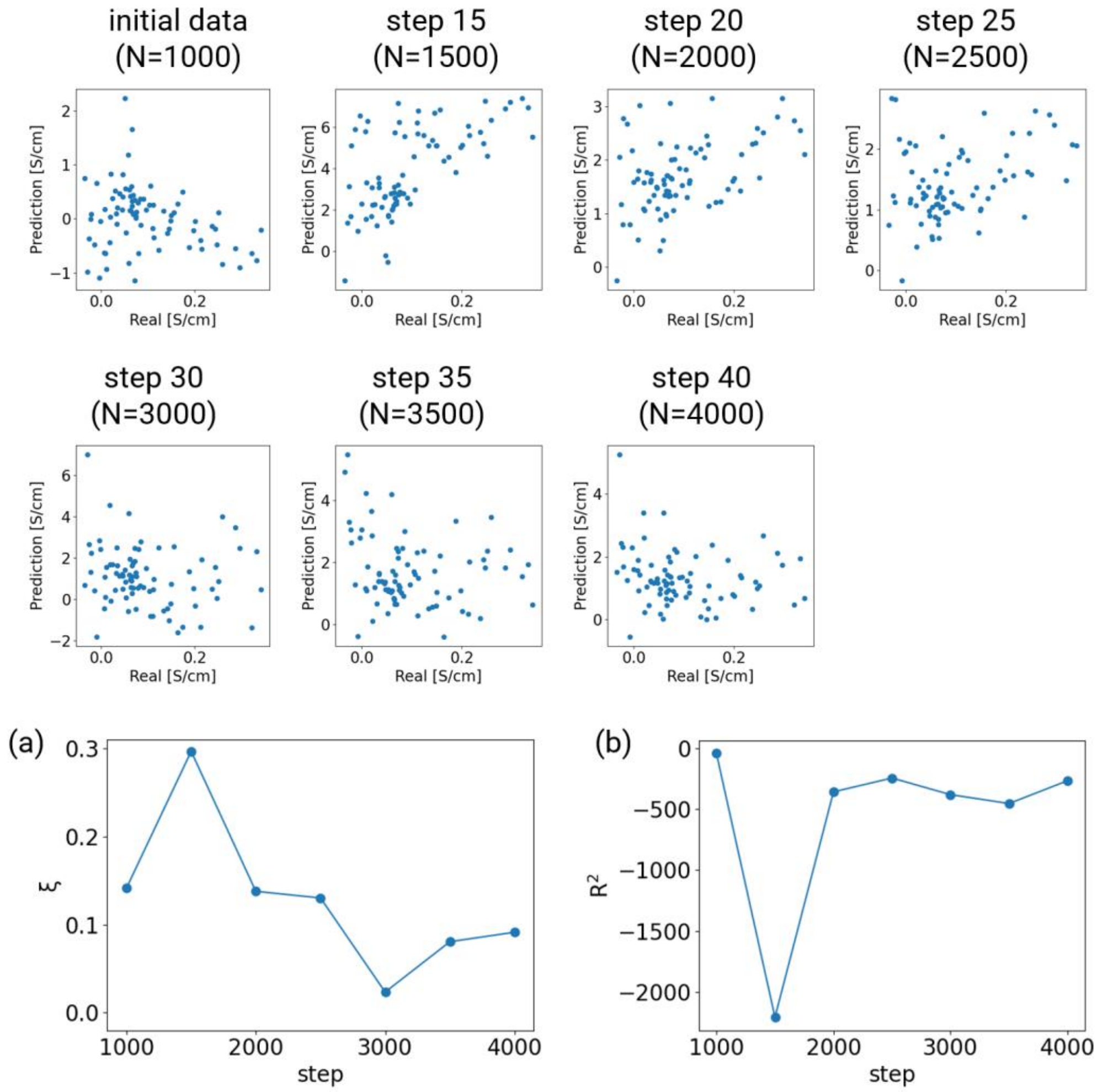

Figure S11 (Upper) Scatter plots of the KMC-calculated (Real) versus FM-predicted conductivities depending on the iteration steps. The prediction model is factorization machine (FM), and *N* is the number of training data. (Lower) Prediction accuracy depending on the iteration steps, evaluated using Chatterjee's rank-based coefficient (a) and R^2 value (b).

Supporting Note G: Analysis of structure–conductivity relationship

Using Random Forest regression, we investigated the relationship between dopant configurations and ionic conductivity. The structure data were converted into the structural descriptors defined in our previous paper[1]. The training (N=8,720) and test (N=1,000) datasets were selected randomly from the dataset (N=40×3×81) obtained from the optimizations reported in Figure 2(b) of the main text. Figure S12 shows scatter plot of the KMC-calculated (Real) versus predicted conductivities for the test data, and Figure S13-S15 show the importance of each descriptor. These results indicate that the configuration close to the interfaces is important to describe ionic conductivity.

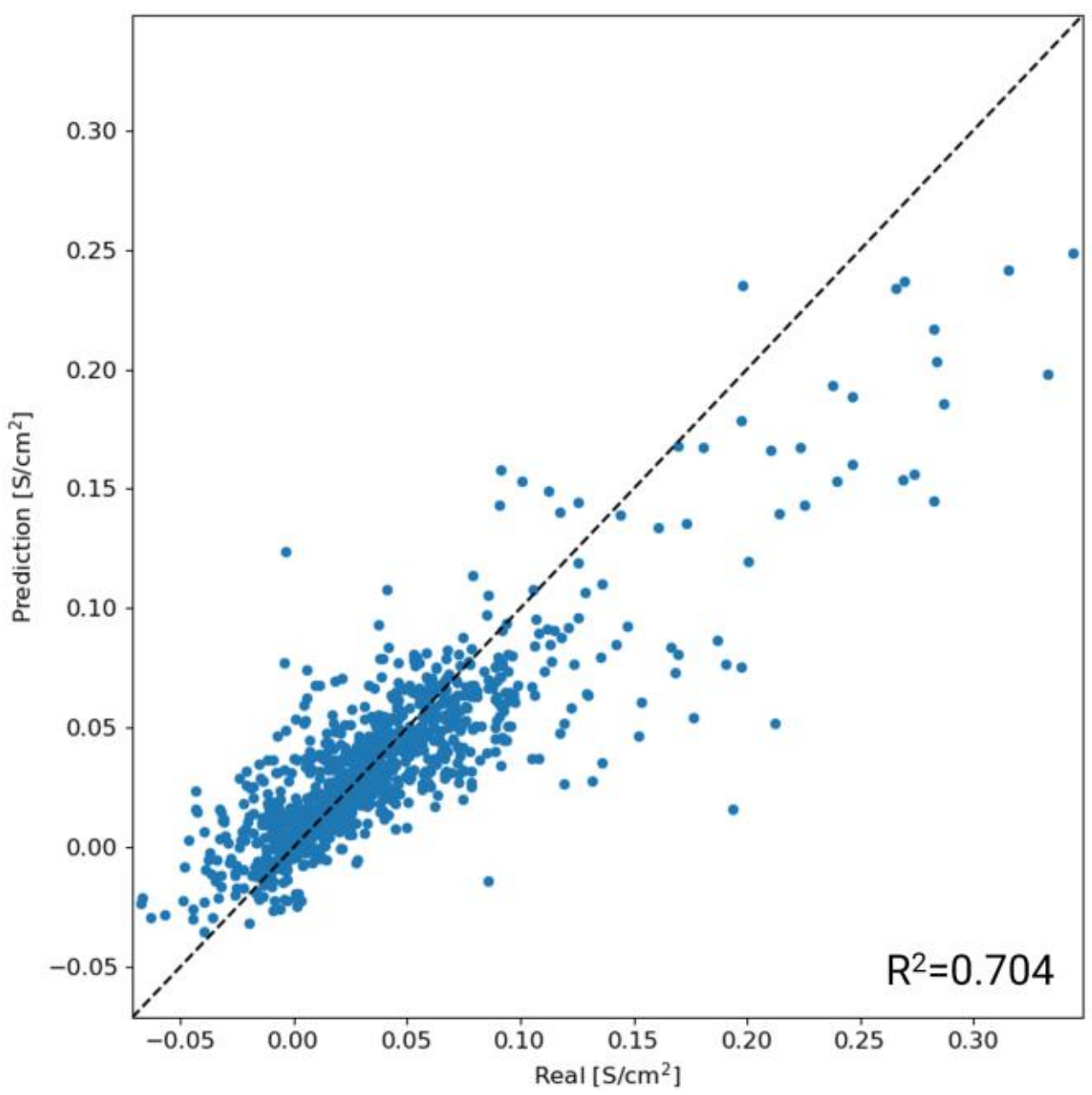

Figure S12 Scatter plot of the KMC-calculated (Real) versus predicted conductivities using Random Forest regression.

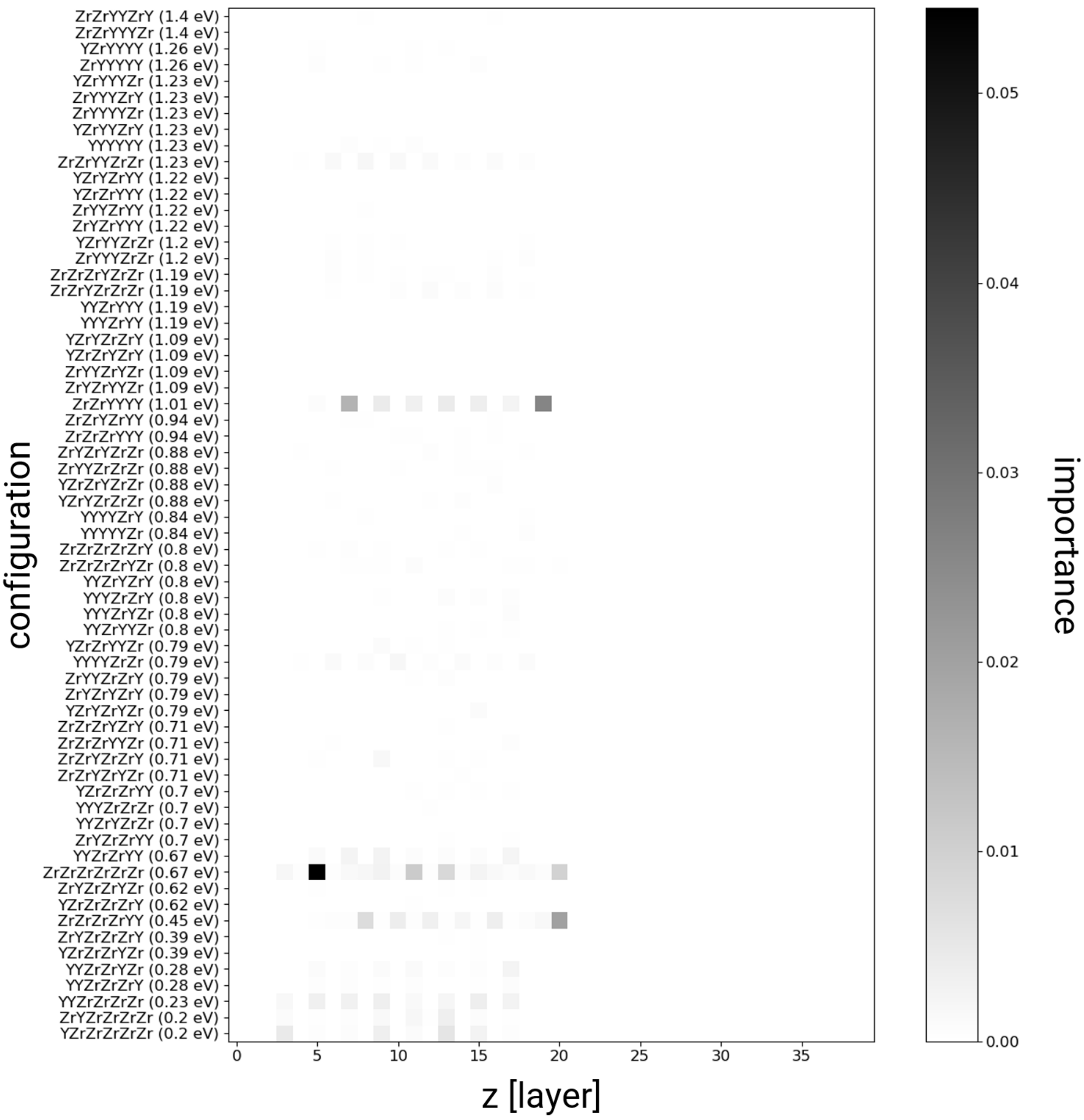

Figure S13 Importance of structural descriptors, $h_m^{-z}(z)$.

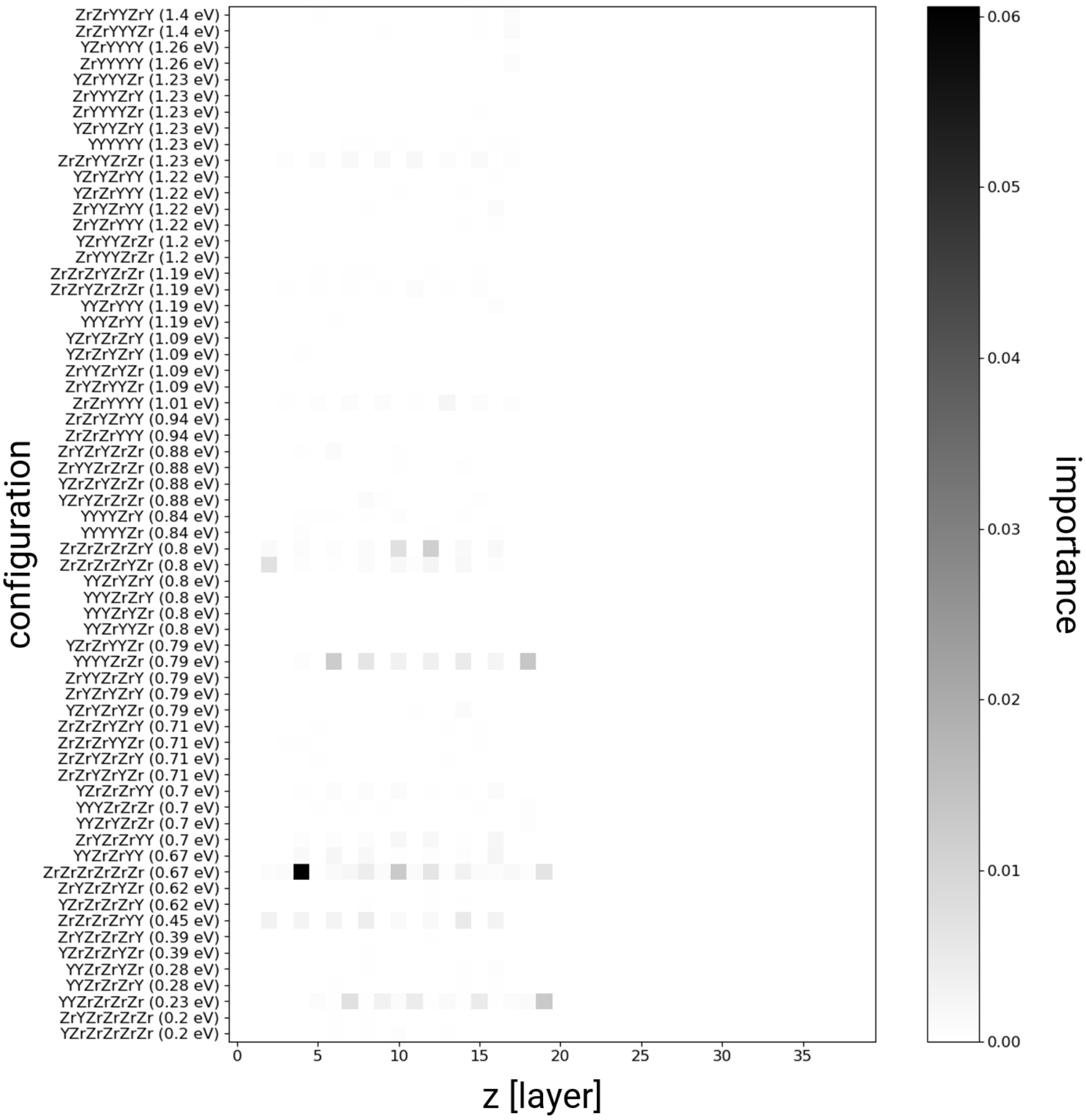

Figure S14 Same for Figure S13, but for $h_m^{+z}(z)$.

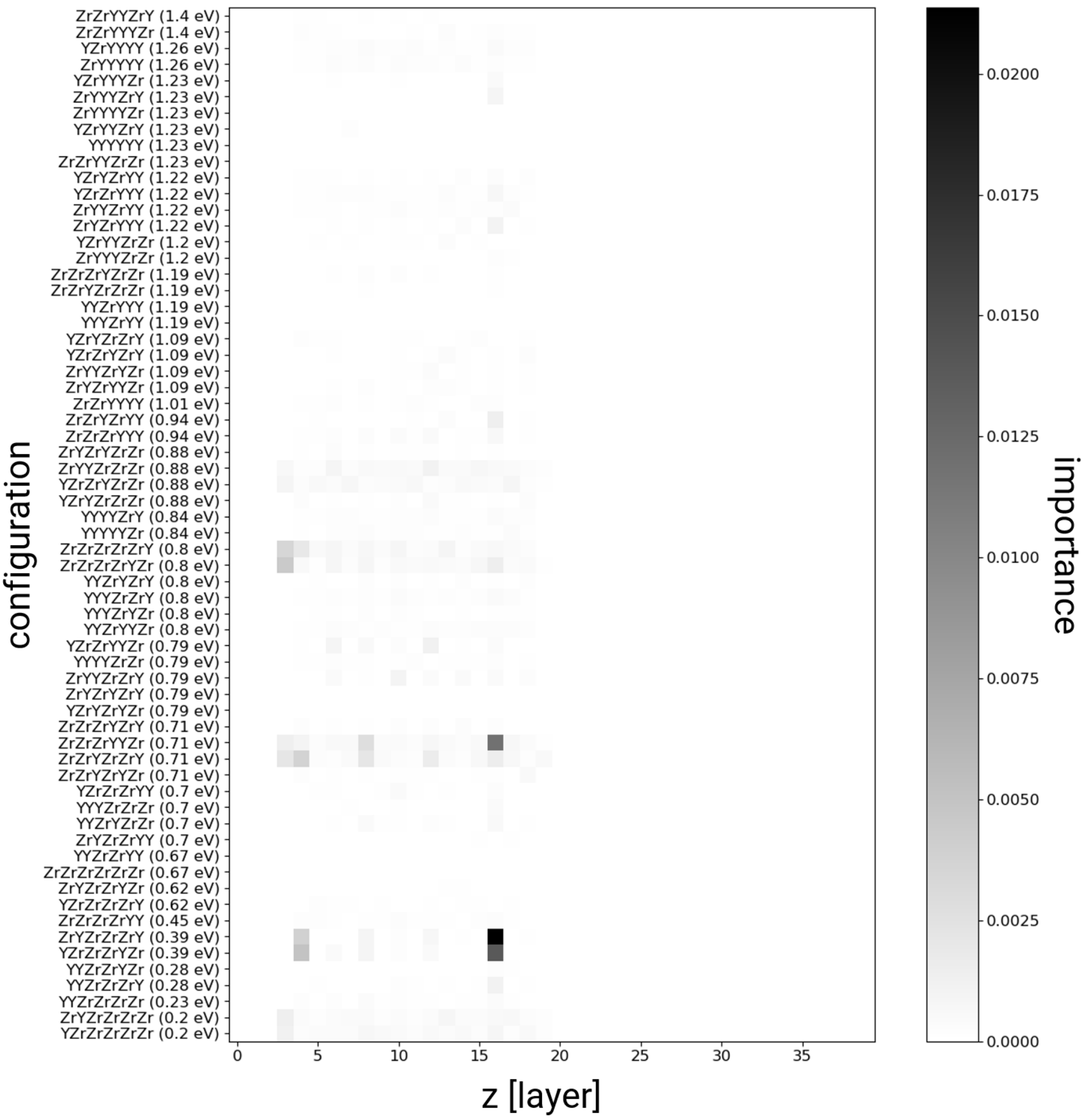

Figure S15 Same for Figure S13, but for $h_m^{\mathrm{xy}}(z)$.

Supporting Note H: Analysis of the electrostatic contribution

In Supporting Note G, we investigated the relationship between dopant configuration and conductivity, suggesting that the configuration close to the interfaces is important for high conductivity as also found in Figure 3 of the main text. Thus, we investigated the details of the system by comparing the high-conductivity configuration with the configuration whose conductivity is close to the experimentally reported value, visualized in Figure 3 of the main text. Figure S16 compares the systems in terms of the Y distribution (a, b), averaged vacancy distribution (c, d), and averaged electrostatic potential (e, f) along the z axis (Equilibration: 1,000 steps, Sampling: 8,000,000 steps run and sampling every 10,000 steps, i.e., N =800). Comparing panels (c) and (d), we found enhanced accumulation of oxygen vacancies at the interfaces in the high-conductivity case than in the random configurations. As a result, a significant difference is observed in the electrostatic potential profile as shown in panels (e) and (f). The layered dopant configurations promote oxygen vacancy accumulation along the layers, which modifies the electrostatic contributions to the activation barriers and may thereby facilitate ionic transport. Thus, we investigated the electrostatic contribution for the activation barrier. Figure S17-19 show the ion-hop activation energy barrier profiles along the z axis for the representative systems. As a result, the electrostatic energy contribution, ΔE, is much smaller than the configuration-dependent migration barrier, and high energy barriers were found around the interfaces in the high-conductivity configuration, even though such high barriers would generally be expected to hinder ion migration. Despite these analyses, a definitive explanation for the observed enhancement in conductivity remains elusive. In this study, we conclude that further investigation, such as examining systems with different vacancy concentrations, is needed to understand the origin of the enhanced ionic

conductivity.

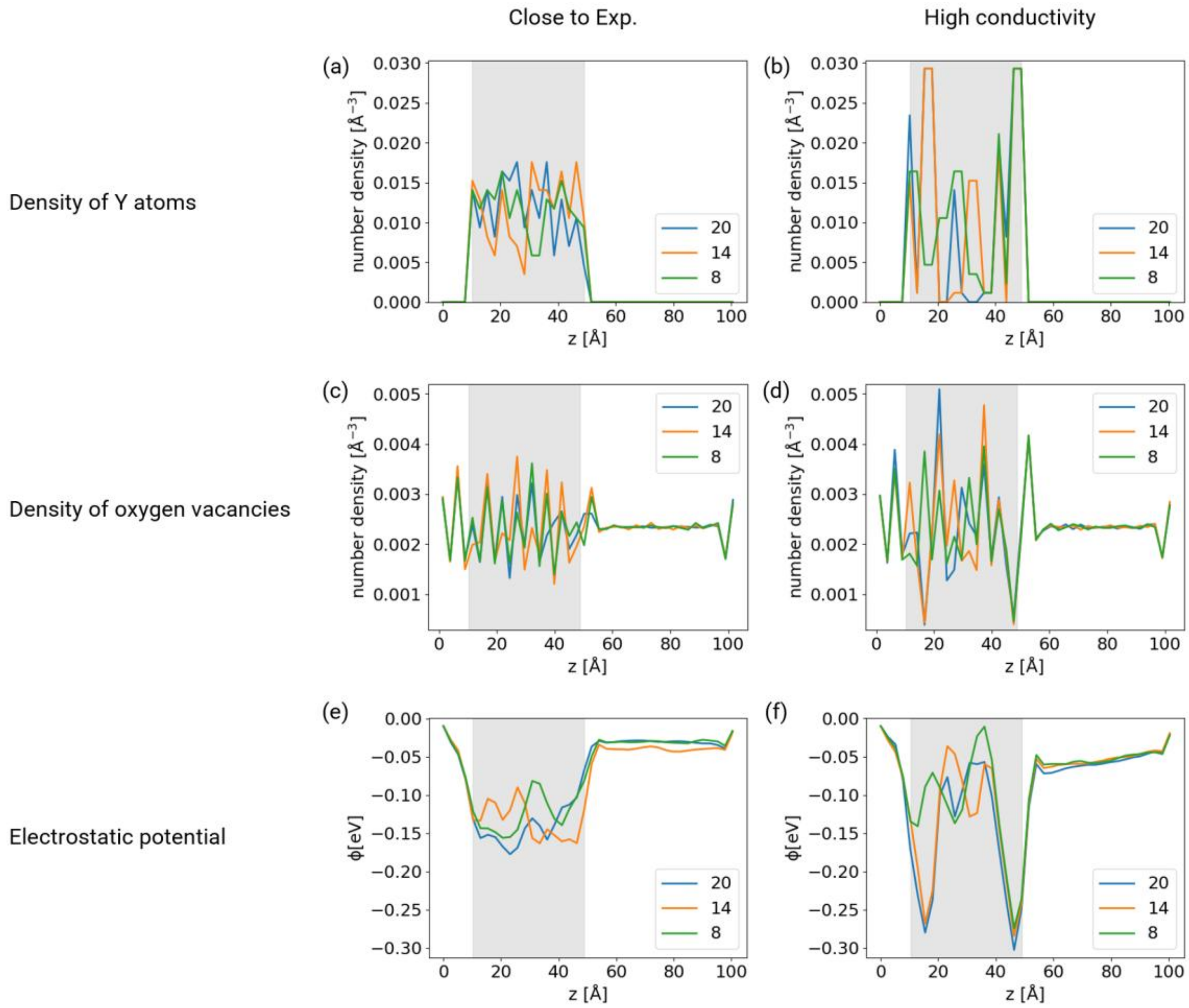

Figure S16 Comparison of systems with conductivity close to the experimentally reported value and those with high conductivity. Blue, orange, and green correspond to the block composition index *b*=20, 14, and 8, respectively. The gray region represents the YSZ layers.

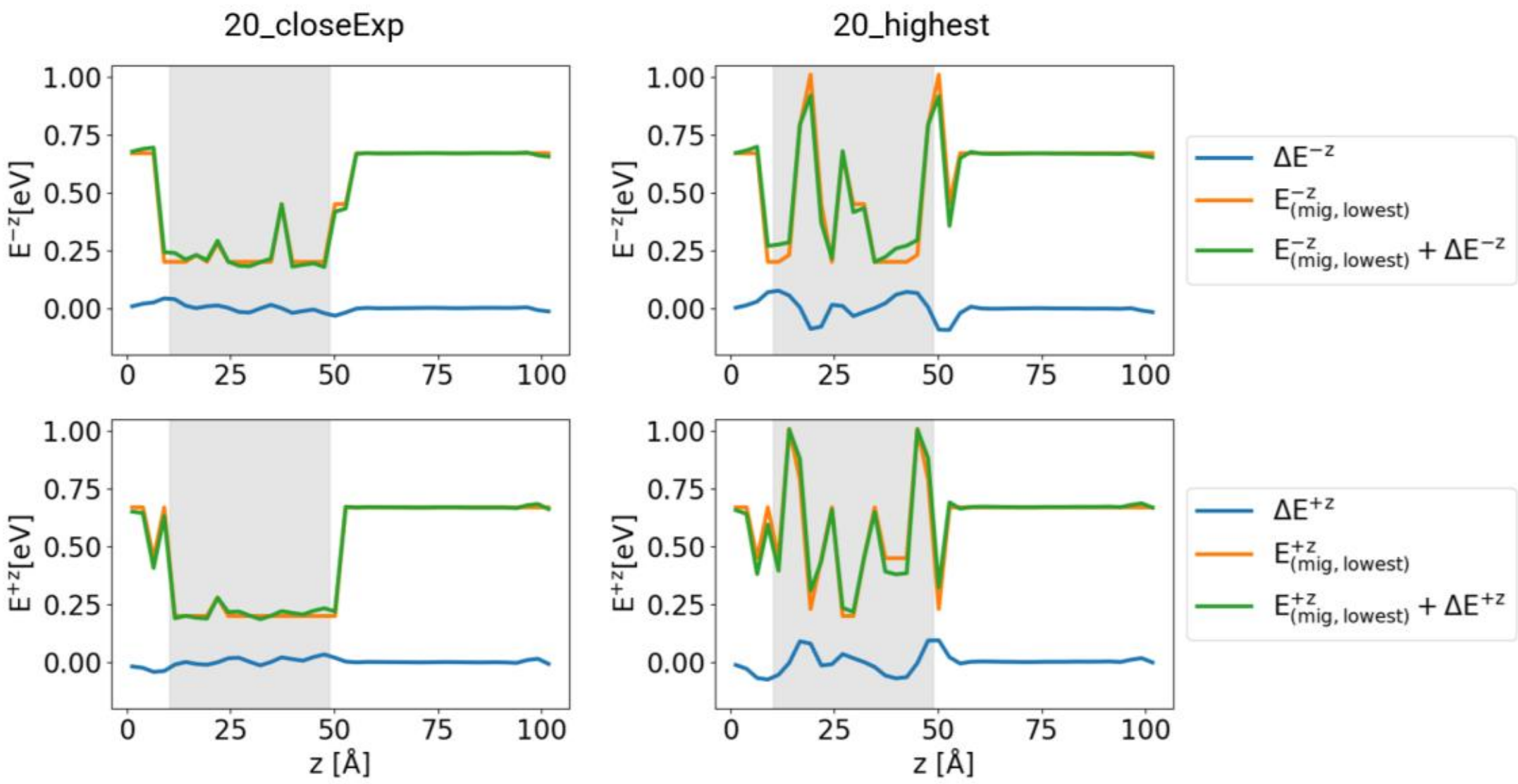

Figure S17 Ion-hop activation energy barrier profiles along the z axis for b=20. Upper panels show the energy for hopping in the -z direction, whereas bottom panels show the energy in the +z direction. ΔE is the electrostatic energy contribution, and $E_{(\mathrm{mig,lowest})}$ is the lowest configuration-dependent migration energy at each z. The left column shows the system with conductivity close to the experimentally reported value, whereas the right column shows the system with high conductivity.

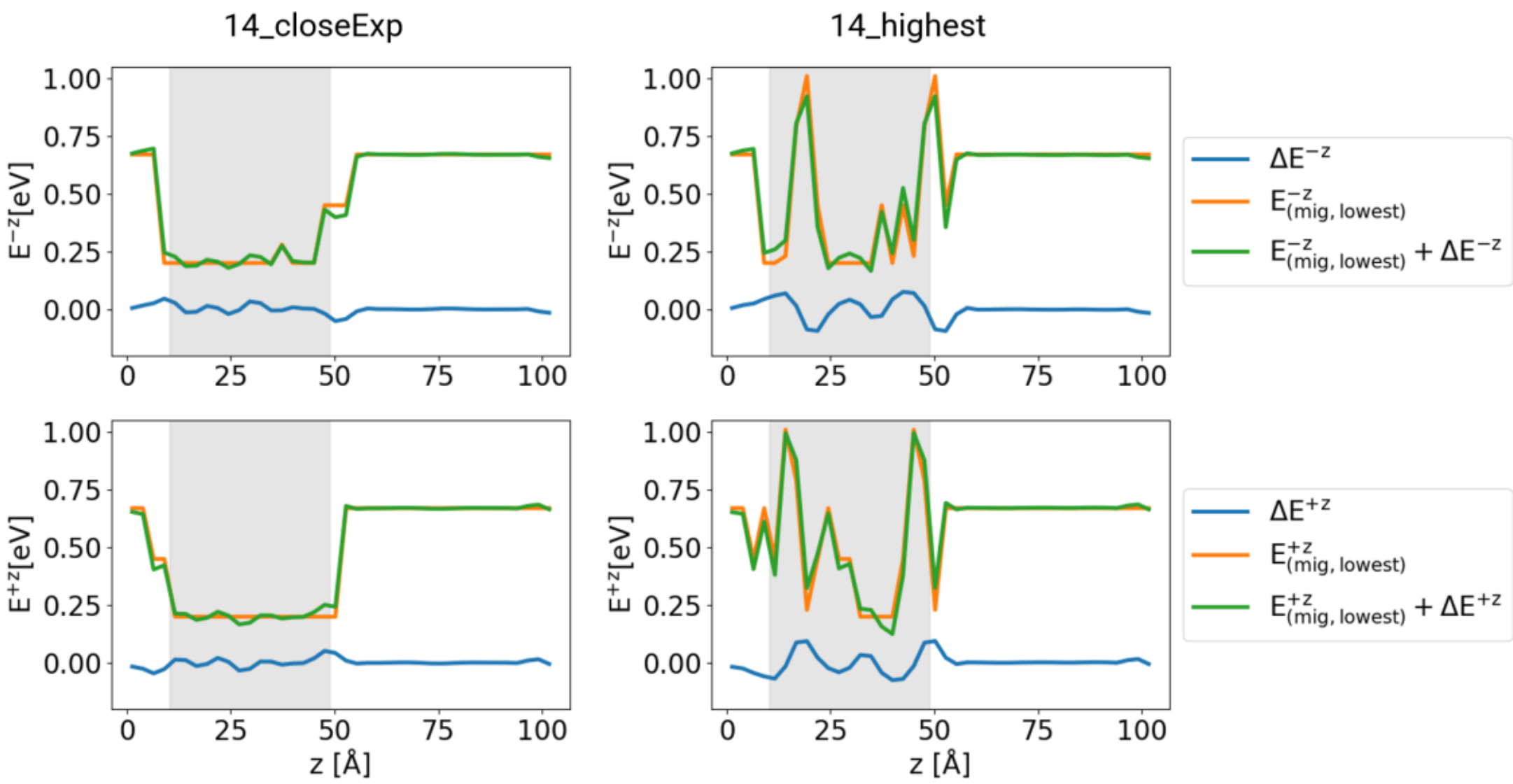

Figure S18 Same as Figure S17, but for b=14.

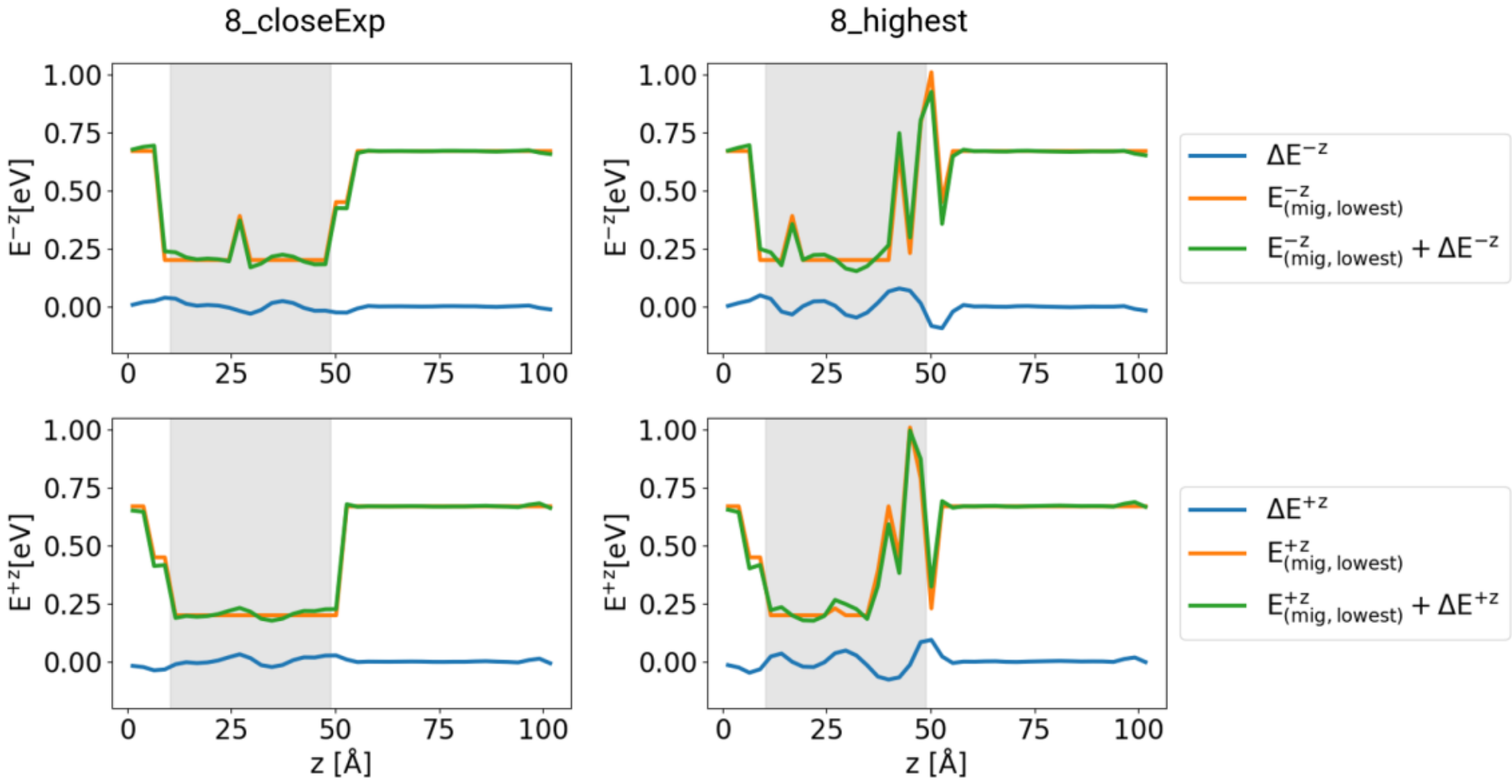

Figure S19 Same as Figure S17, but for *b*=8.

References

(1) Koizumi, A.; Ivonina, M.; Tamura, R.; Tada, T. Linking Microscopic Structures to Ionic Currents in Solid Electrolytes via Kinetic Monte Carlo and Regression Analysis. *J. Phys. Chem. C* **2026**, *130* (10), 3897–3908. https://doi.org/10.1021/acs.jpcc.5c06852.

(2) Pornprasertsuk, R.; Ramanarayanan, P.; Musgrave, C. B.; Prinz, F. B. Predicting Ionic Conductivity of Solid Oxide Fuel Cell Electrolyte from First Principles. *J. Appl. Phys.* **2005**, *98* (10).

(3) Grieshammer, S.; Eisele, S.; Koettgen, J. Modeling Oxygen Ion Migration in the CeO_2 –ZrO_2 – Y_2 O_3 Solid Solution. *J. Phys. Chem. C* **2018**, *122* (33), 18809–18817. https://doi.org/10.1021/acs.jpcc.8b04361.

(4) Yamahara, K.; Jacobson, C. P.; Visco, S. J.; De Jonghe, L. C. Influence of Powders on Ionic Conductivity of Polycrystalline Zirconias. *ECS Proc. Vol.* **2003**, *2003* (1), 187.

(5) Chatterjee, S. A New Coefficient of Correlation. *J. Am. Stat. Assoc.* **2021**, *116* (536), 2009–2022. https://doi.org/10.1080/01621459.2020.1758115.
